# Deep atrous context convolution generative adversarial network with corner key point extracted feature for nuts classification

**DOI:** 10.1038/s41598-026-36238-2

**Published:** 2026-01-27

**Authors:** M. Shyamala Devi, M. Jaiganesh, S. Priya, E. Elakkiya

**Affiliations:** 1 National Satellite Information Research Institute, Kyungpook National University, 80 Daehak-ro, Buk-gu, Daegu, 41566 Korea; 2 Department of Robot and Smart System Engineering, Kyungpook National University, 80, Daehak-ro, Buk-gu, Daegu, 41566 Republic of Korea; 3 Department of Information Technology, Karpagam College of Engineering, Coimbatore, Tamil Nadu India; 4 Manipal Institute of Technology Bengaluru, Manipal Academy of Higher Education, Manipal, India; 5 Department of Computer Science and Engineering, SRM University, Amaravati, 522240 Andhra Pradesh India

**Keywords:** Accuracy, Atrous convolution, Augmentation, Classification, Context block, Corner key point, GAN, Deep learning, Feature extraction, Computational biology and bioinformatics, Engineering, Mathematics and computing

## Abstract

Deep learning-based nut classification has emerged as a viable way to automate the detection and categorization of different nut varieties in the food processing and agriculture sectors. Conventional techniques for classifying nuts mostly rely on manually created characteristics like texture, color, shape, or edges. These characteristics frequently fall short of capturing the image’s complete complexity, particularly when nuts show tiny visual variances. This research proposes Deep Atrous Context Convolution Generative Adversarial Network (DAC-GAN) model that categorize the 8 classes of nuts like brazil nuts, cashew, peanut, pecan nut, pistachio, chest nut, macadamia and Walnut. This research uses Common Nut KAGGLE dataset with 4,000 nuts images of 8 nuts classes. The DAC-GAN approach overcomes the difficulties of having limited labelled data for nut classification tasks by employing DCGANs’ ability to produce high-quality, synthetic nut images to supplement the dataset. The DCGAN comprises of a discriminator and a generator block. The discriminator block develops the ability to differentiate between synthetic and real images, while the generator block generates realistic nut images from random noise. The real images along with the DCGAN generated images are processed with feature filtering methods to extract the Corner Key Points Featured (CKPF) nuts images. To further enhance the feature selection, the CKPF edges are extracted from the image that provides unique, geometrically distinctive critical corners to further process for representative learning. To proceed with the effective feature extraction and model learning, the CKPF nuts images are processed with atrous convolution that capture the intricate details by expanding the receptive field without losing resolution. The novelty of this work exists by appending the filtration and atrous convolution that acquire the spatial data features from the nut’s images at various resolutions. Atrous convolution was refined by appending the pre-context and post-context block that add the image level information to the features. The effectiveness of the DAC-GAN model was validated with the traditional augmented dataset with all existing filtering images and CNN models. Implementation outcome shows that DAC-GAN found to exhibit high accuracy of 99.83% towards the nuts type classification. The superiority of the DAC-GAN method over traditional approaches is demonstrated by extensive experiments on augmented and DCGAN generated datasets, which achieve higher classification accuracy and generalization across a variety of nut type categorization. The outcome demonstrates that the DCGAN together with atrous convolution have the potential to be an effective tool for automating nut sorting in food industry.

## Introduction

In the agricultural and food processing sectors, automated nut classification is essential for effective sorting, quality assurance, and marketing. However, precision, effectiveness, and resilience often pose problematic with classic nut classification techniques, particularly when working with large amounts of data or a variety of different conditions. Deep learning-based methods have demonstrated significant potential in addressing these issues because of their capacity to automatically extract intricate patterns and characteristics from unprocessed data. In recent years, the GAN was used for generating unlimited synthetic data that automate complex feature learning and pattern from the unprocessed data. Depending on the lighting, background, perspective, and orientation, nut images can show a lot of variation. Conventional approaches frequently fall short in these situations, rendering them unsuitable for practical use as it involves manual interpretation for feature extraction. With this motivation, this research proposes DAC-GAN that integrates the feature filtering methods by corner key point extraction and atrous convolution at the end to finetune the GAN model.

The proposed model DAC-GAN uses DCGAN for augmenting the data that contains generator and discriminator block. For applications requiring image generation or data augmentation, the DCGAN architecture offers an effective method for synthesizing realistic images. DCGANs learn to produce images with intricate spatial relationships and excellent quality by utilizing convolutional layers. The effectiveness and stability of downstream classification models can be enhanced in applications like nut classification by using DCGANs to produce synthetic nut images to supplement training data. The DCGAN generated images to processed to form filtering images. Additionally, corner key point discrimination performs well for identifying essential visual characteristics in images, including edges or corners, which are crucial for differentiating between different kinds of nuts. The model can improve classification accuracy by prioritizing the most discriminative aspects of the image by concentrating on these crucial elements. The filtered images are further processed with atrous convolution by allowing for an expanded receptive field. The atrous convolution allows the model to collect additional spatial information without significantly increasing computing complexity. This method can greatly enhance the model’s capacity to identify contextual elements and fine-grained elements in nut images, including size, texture, and shape all of which are critical for precise classification. By combining cutting-edge approaches for feature extraction, data augmentation, and spatial context analysis, this DAC-GAN model seeks to overcome the drawbacks of conventional approaches.

### Paper organization

The broad arrangement of this article’s structure was as follows. Section 1.2 highlights the contributions of the research study. Section 2 reviews the inferences drawn from the literature about the classification of nuts. Section 3 presents the suggested DAC-GAN model research technique. Section 4 examines the mathematical modelling of the suggested DAC-GAN. Section 5 reports the suggested DAC-GAN’s execution outcomes. Section 6 completed the proposed DAC-GAN model with the results and suggestions for improvement.

### Contributions of this research

The following are the main contributions of this research are three-folded.

**Fig. 1:**
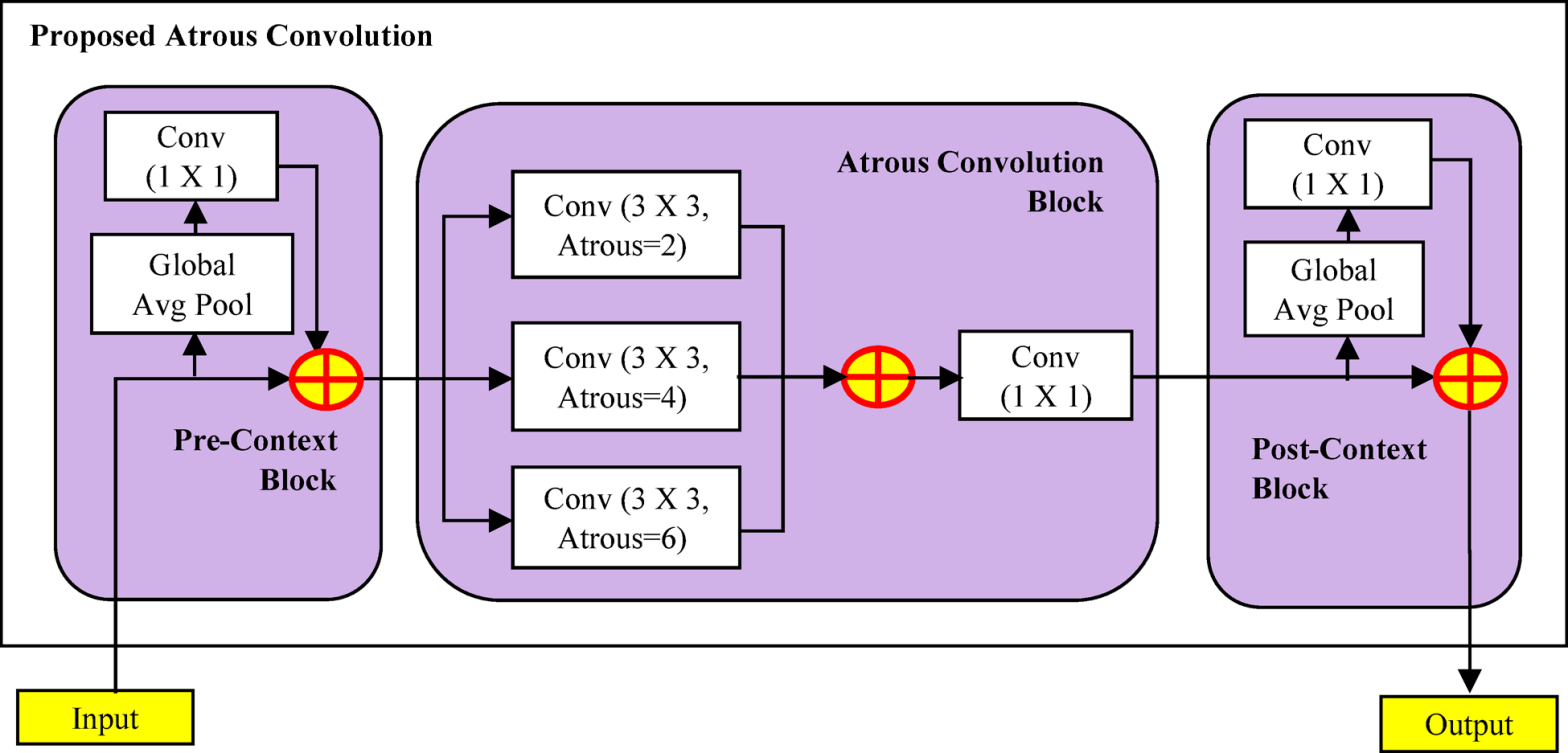
Atrous context convolution block structure.

(i) For addressing the difficulty of having limited labelled data, the DCGAN was used to perform data augmentation of nuts images as the initial contribution. The adaptation of DCGAN-based data augmentation method increases the resilience and effectiveness of the classifier by offering a variety of nut image variations, that facilitates capacity to generalize.
(ii) The Second contribution provides efficient data preprocessing through feature selection, that focus on filtering the edge details from nuts images by generating CKPF nut images.
(iii) The third contribution is the design of proposed DAC-GAN. This hybrid DAC-GAN model was designed with the integration of DCGAN, corner key point extraction followed by atrous convolution. The existing Atrous convolution was refined by appending the pre-context and post-context block that add the image level information to the features as in Fig. 1.

## Background study

Deep learning has shown itself to be an effective method for classifying nuts automatically, with significant improvement in scalability, accuracy, and speed. The inferences form the literature survey is shown in the Table 1.

**Table 1:** Inferences from the literature review.

**Methodology**	Inference and advantage	Pitfalls
**ML Methods**: • MLP ANN^2^, SVM^7^ for cashew kernel classification. • PCA^3^, Decision tree^11^, PCA^11^ for Almond Classification • SVM^12^, PCA with ANN^16^, Random forest^33^ for nut classification • Gradient boosting^20^, random forest^20^, ANN with discriminant analysis^21,24^ for hazelnut, chestnut^29^ SVM^23^ for pinenut classification	• Achieved up to 93–94% accuracy for various nut types. • Lightweight and efficient for smaller datasets. • Suitable for feature-based classification with structured data.	• Require labeled data and sensitive to imbalance. • Limited adaptability to new or noisy data. • Lack automatic feature extraction.
**Image Processing Methods**: • Otsu thresholding^4^ for Almond Classification • Improves KNN^13^, Otsu method^19^, Slime Mould Algorithm^28^ for pistachio nut classification • Big transfer model^25^ for hazelnut classification • KMeans++^34^, SVM + HOG^39^ for nuts type, areca nuts^36,38^ Classification.[^35^]	• Achieved 85–97% accuracy depending on dataset. • Enable extraction of texture, color, and shape features. • Effective for preprocessing and visual feature isolation.	• Dependent on image quality and lighting. • Sensitive to noise and orientation. • Limited scalability for large datasets.
**CNN Methods**: • Inception-V3^1^, ResNet50^1^, VGG-16^1^, YoloV5^5^, Sequential CNN^37^ for cashew kernel classification. • Sequential CNN^4,8^, conv2D CNN^6^, optimized Deep CNN with flower pollination algorithm^9^, DenseNet^10^, EfficientNetB0^10^, MobileNet^10^, MobileNet V2^10^, NASNetMobile^10^ for Almond Classification • AlexNet^14,27,31,40^, VGG16^14,16,27,30,31,40^, ResNet20^15,16,18,27,32,40^, InceptionV3^30–32,40^, DenseNet^15,40^, Adaptive CNN^41^ for pistachio nut, peanut classification • EfficientNet^17^ and InceptionV3^17^, ResNet50 with feature reduction^18^, DL4J feedforward^20^, Sequential CNN^22^, AlexNet^26^ for hazelnut kernels classification.	• Ac^35^hieved 94–99% accuracy across datasets. • Capable of automatic feature learning and multi-level representation • Highly generalizable with augmentation and transfer learning.	• Require large datasets and high computation. • Prone to overfitting with small data. • Sensitive to hyperparameter settings.

DL approaches are enabling more accurate and efficient nut classification because of developments in model architectures, data augmentation, and transfer learning^1,3^. However, to properly utilize deep learning in this industry, issues including class imbalance, real-time processing, and dataset quality must be resolved. The machine learning methods like SVM, random forest^12,20^ have been implemented for the classification of various type of nuts like peanut, pistachio and chest nut. The Artificial neural network has integrated with multi-layer perceptron^2,11^ for the nuts type classification. The image processing methods also integrated with KNN, KMeans^13,19^ clustering methods for classification of nuts type. The Otsu thresholding^4^ methods also were used for the nuts type classification. The pretrained CNN and sequential CNN^4,32^ models were implemented towards nuts type classification. From the inferences made form the review work, still there remains a challenge towards data augmentation and filtering methods towards nuts type classification. The proposed DAC-GAN have addressed the issue of data augmentation by creating synthetic images from DCGAN and the filtering methods by extracting the corner key point features from nuts image.

## Materials and methods

### Dataset collection and distribution

The proposed DAC-GAN model was developed and evaluated using a nut image dataset consisting of eight distinct nut classes, namely Brazil nut, Cashew, Chestnut, Peanut, Pecan nut, Pistachio, Macadamia, and Walnut. The dataset was sourced from the publicly available Common Nut Dataset on Kaggle^42^, which provides a diverse collection of high-quality nut images under varying lighting conditions, orientations, and backgrounds. A total of 4,000 images were utilized, with 500 images per class to ensure balanced class representation. To facilitate robust model training and reliable performance evaluation, the dataset was divided into training, validation, and testing subsets following a standard data splitting strategy. The dataset distribution of the Common Nut dataset is shown in Table 2. Specifically, 20% (800 images) of the dataset was reserved exclusively for testing to ensure unbiased evaluation of the model’s generalization capability, while the remaining 80% (3,200 images) were used for augmentation. The augmentation was subsequently performed using the proposed DAC-GAN model. In this process, the augmentation generates 21 images for each dataset image.

**Table 2:** Dataset distribution for common nut dataset.

Nut class	Data distribution						
	Actual	Testing	Actual after testing	Augmentation	Actual after Augmentation	Train	Validation
Brazil nut	500	100	400	8400	8800	7040	1760
Cashew	500	100	400	8400	8800	7040	1760
Chestnut	500	100	400	8400	8800	7040	1760
Peanut	500	100	400	8400	8800	7040	1760
Pecan nut	500	100	400	8400	8800	7040	1760
Pistachio	500	100	400	8400	8800	7040	1760
Macadamia	500	100	400	8400	8800	7040	1760
Walnut	500	100	400	8400	8800	7040	1760
**Total**	**4000**	**800**	**3200**	**67,200**	**70,400**	**56,320**	**14,080**

To eliminate any class imbalance, the dataset was designed to maintain equal representation across all eight nut categories, with 500 original images per class. The DAC-GAN augmentation process generated an additional 8,400 synthetic images per class, increasing each category to 8,800 samples. This uniform expansion preserved data balance throughout the training, validation, and testing phases, ensuring that the model learned representative features from all classes equally and avoided classification bias. The augmentation results in 67,200 ending with 70,400 total images after augmentation. The training set comprised 80% (56,320 images) of the total data, and 20% (14,080 images) was allocated for validation to fine-tune the model’s hyperparameters and prevent overfitting. This strategy ensured strict separation between the testing and augmented data, providing a reliable assessment of the model’s generalization capability.

### Proposed DAC-GAN research methodology

**Fig. 2:**
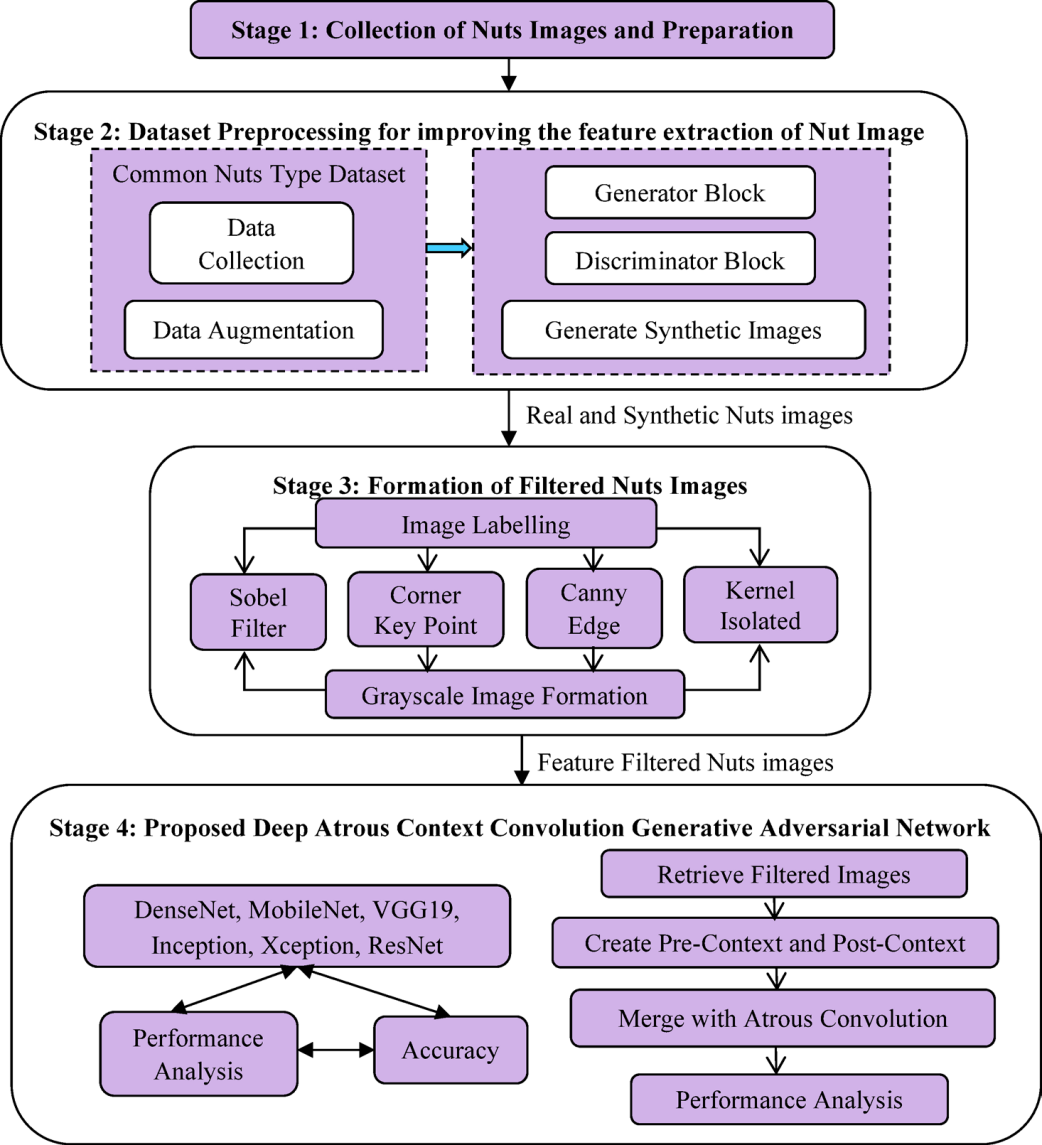
Proposed DAC-GAN research methodology.

The proposed DAC-GAN model was designed to classify 8 classes of nuts like brazil nuts, cashew, peanut, pecan nut, pistachio, chest nut, macadamia and walnut by using Common Nut KAGGLE dataset^42^ with 4,000 nuts images of 8 nuts classes. The DAC-GAN research methodology is shown in Fig. 2. The stage 1 of the DAC-GAN starts with collection of the nut’s images comprising the 8 classes of nuts images.

**Fig. 3:**
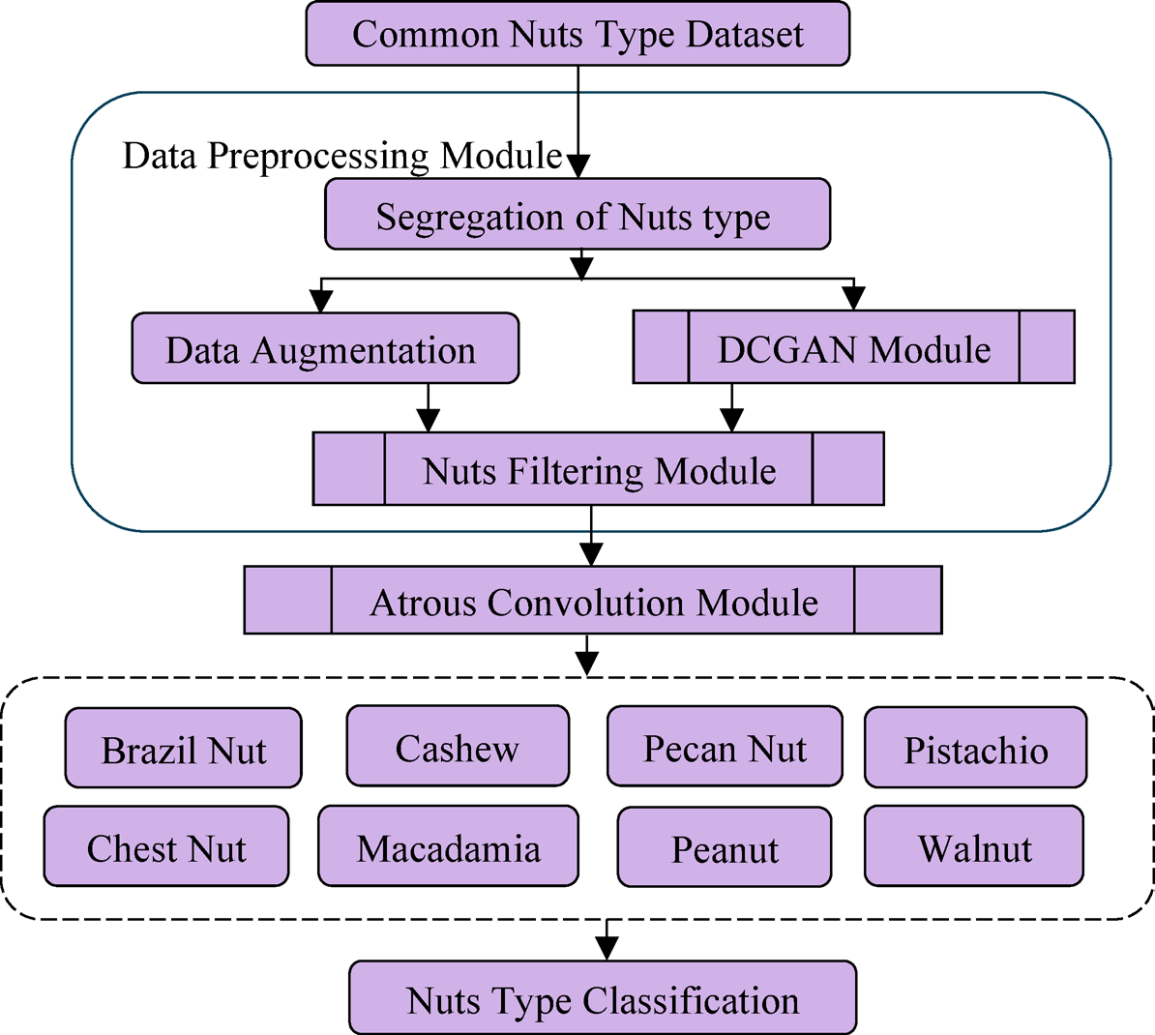
Overall workflow of DAC-GAN.

The Stage 2 focus on grouping, labeling, augmenting and processing the generation of synthetic images using DCGAN. A well-defined data management method was adopted to ensure both model reliability and fairness during evaluation. Initially, 20% of the dataset (800 images) was held out as the test set, consisting solely of authentic, unaltered images. This portion remained isolated throughout the entire training and augmentation process to prevent data leakage and to ensure an unbiased assessment of the model’s generalization performance. The remaining 3,200 images (80%) were designated for augmentation and model training. The augmentation of 3200 images results in 67,200 images resulting in 70,400 actual images. The augmented dataset was then divided into training and validation subsets to optimize learning and prevent overfitting. Specifically, 80% (56,320 images) were allocated for training, while 20% (14,080 images) were reserved for validation to fine-tune the network parameters. This arrangement facilitated stable convergence of the DAC-GAN model and enhanced classification precision. The Stage 3 of the DAC-GAN model retrieves the augmented data and create the filtering nuts images such as Sobel, corner key point, canny edge and kernel isolated filtered nuts images. Stage 4 deals with the design of the proposed DAC-GAN that retrieves the augmented and synthetic filtered images generated by filtration module and fits with the atrous convolution. The existing Atrous convolution was refined by appending the pre-context and post-context block that add the image level information to the features. The augmented and synthetic filtered images are applied with existing CNN and proposed DAC-GAN to assess the performance. The overall workflow of DAC-GAN is shown in Fig. 3 that starts with dataset preprocessing module that groups the nuts based on its type.

**Fig. 4:**
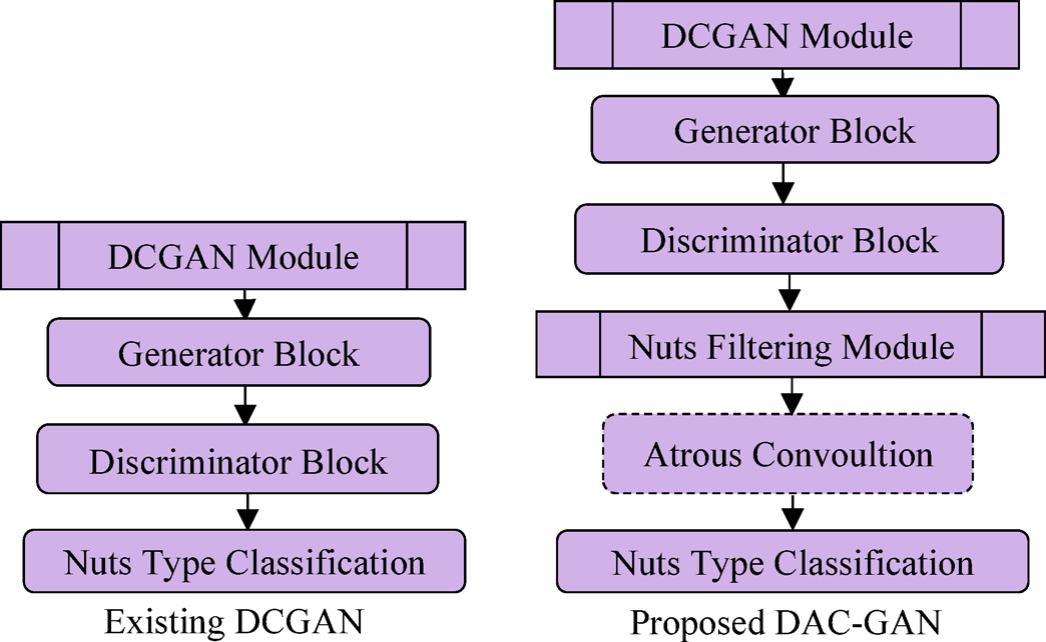
Comparison of Existing DCGAN and proposed DAC-GAN.

**Fig. 5:**
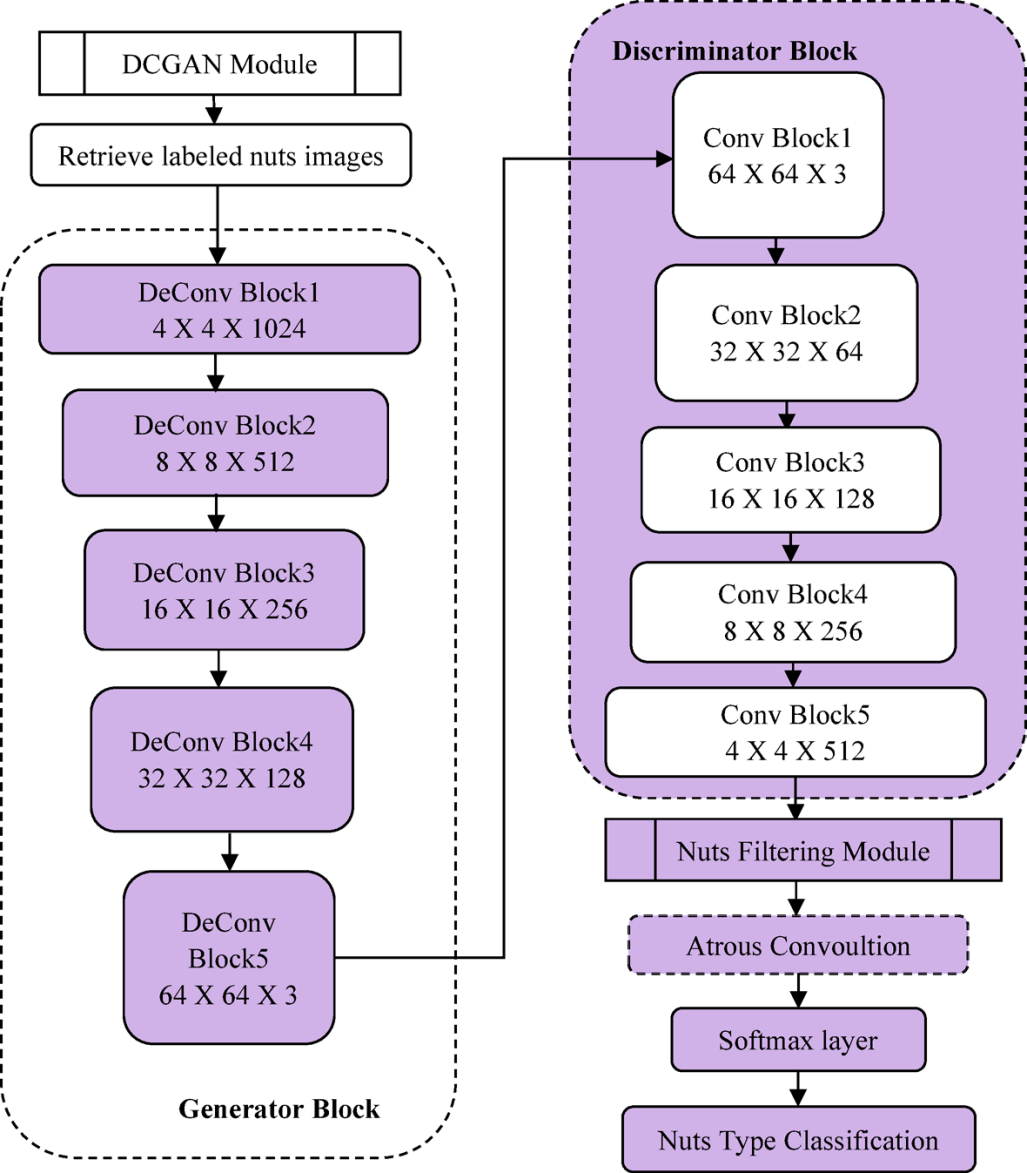
Working process of DC-GAN module.

The labelled nuts images are then processed to form augmented data and processed with DCGAN module that generated synthetic images. The augmented and synthetic filtered images are processed to form the filtered images and then fitted with the atrous convolution to classify the 8 classes of nuts. The comparison of the existing DCGAN and the proposed DAC-GAN is shown in Fig. 4. The proposed DAC-GAN was appended with the nuts filtering module and then followed by processing an atrous convolution. The DCGAN module is shown in Fig. 5 that starts by retrieving the labeled nuts images. The DCGAN contains generator and discriminator block. The generator block learns to generate the images that resemble the dataset. The synthetic images are created by passing the random noise inside the transposed convolution layers for up sampling the input image. The discriminator can distinguish between the real and synthetic images. The generator starts creating more synthetic images based on the number of noise vectors and improve the synthetic image creation with the intent of fooling the discriminator block. The 56,320 training images are processed by the proposed DAC-GAN model.

**Fig. 6:**
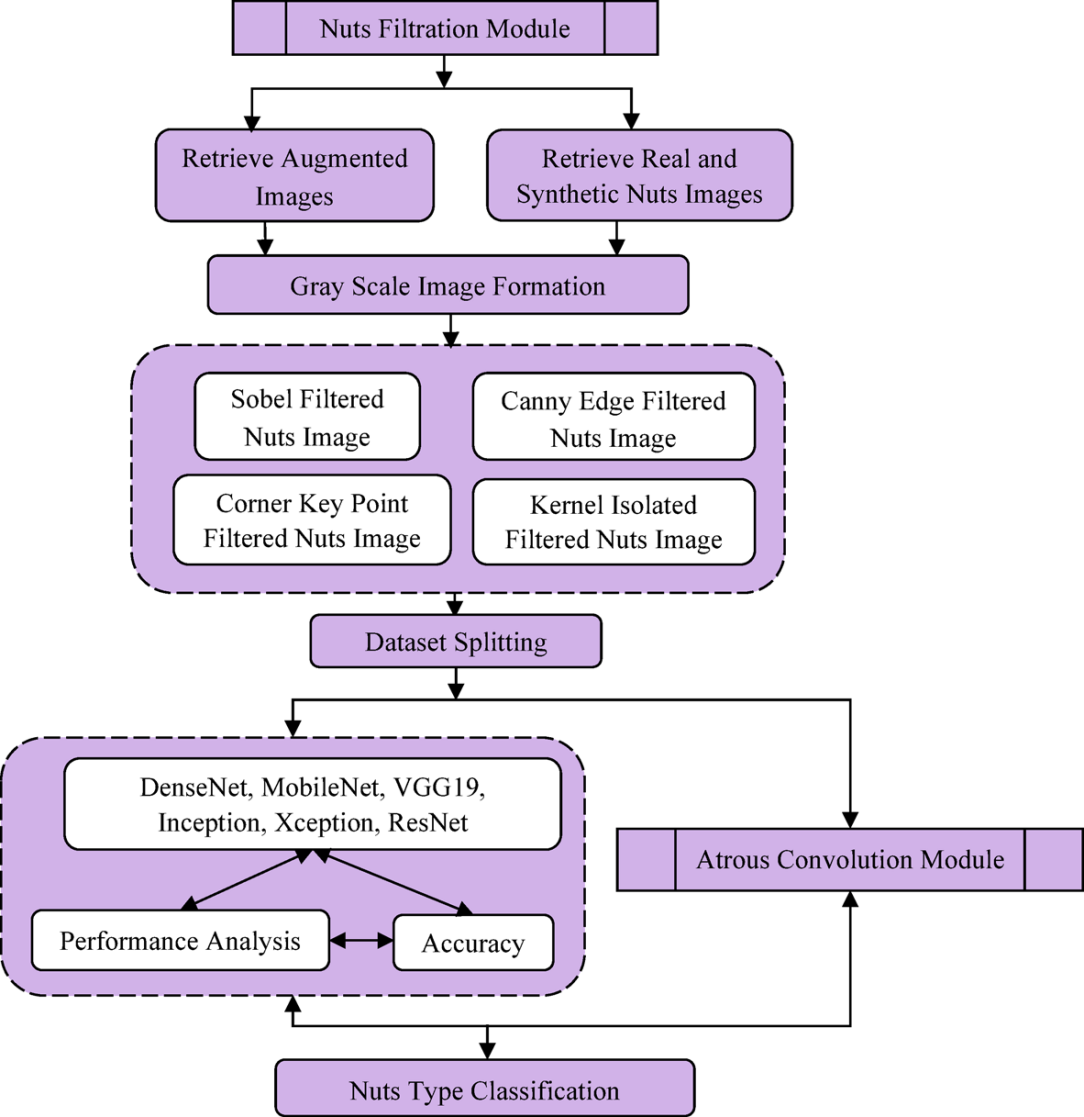
Workflow of Nuts Filtration module.

**Fig. 7:**
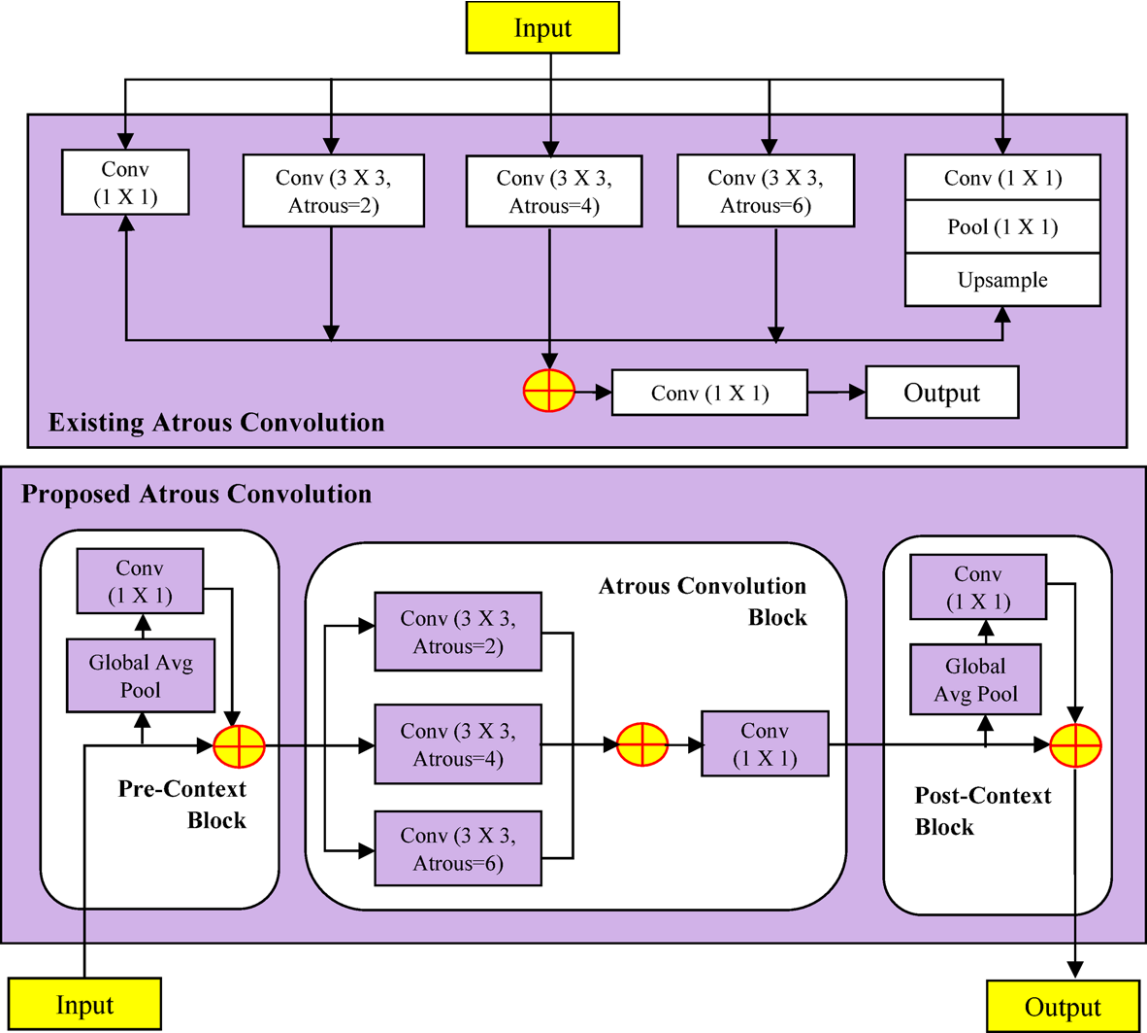
Comparison of existing atrous convolution with proposed atrous context convolution.

Now the augmented and synthetic nuts images are sent to nuts filtration module as shown in Fig. 6 that forms the gray scale images which are processed to form Sobel, corner key point, canny edge and kernel isolated filtered nuts images. The filtered nuts images are processed with atrous context convolution that is shown in Fig. 7 along with the comparison of existing atrous convolution. The normal atrous convolution was refined to append pre-context and post-context block that add the image level information to the features. The pre-context and post-context block have one convolutional block without non-linearity block layer. The feature map (FM) generated by the pre-context block was added with atrous convolution block to generate the atrous FM. The output of the pre-context block is added and sent to the atrous convolution block. The atrous convolution block acquires the spatial data by detecting the fine-grained details at various resolutions. The atrous convolution operation was performed with three consecutive convolutions with atrous rate of 2, 4 and 6 respectively with kernel filter (3 × 3) that increase the kernel size of the filter receptive field without upgrade in the computation parameter. The atrous FM is processed with post-context block and its post-context FM is passed to SoftMax layer with eight neurons to classify eight nuts. The algorithm for DAC-GAN overall framework is shown below.

#### Algorithm

DAC-GAN Model.


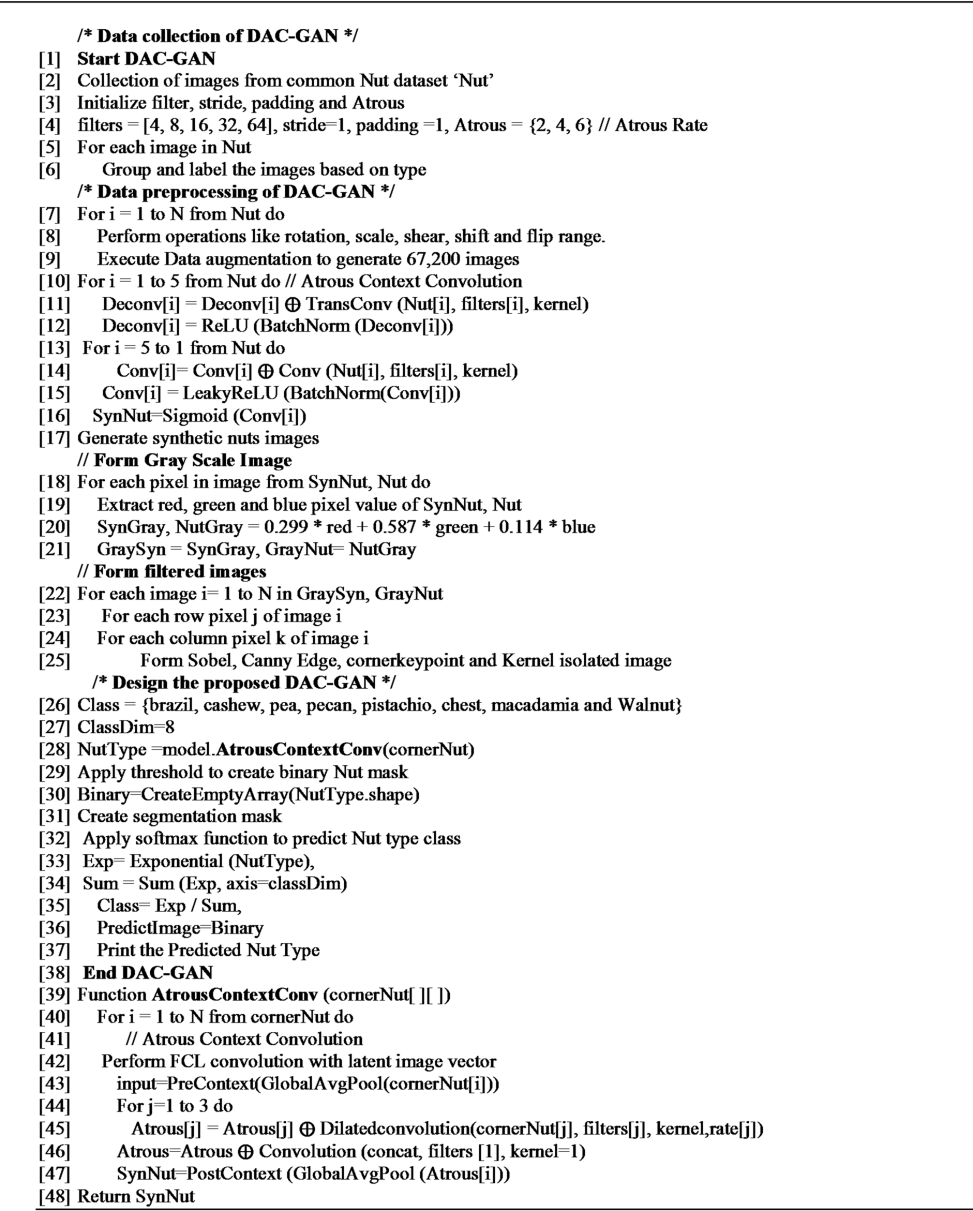


## Mathematical modeling of DAC-GAN

The proposed DAC-GAN model starts with collecting 4,000 images from Common Nut dataset for classifying eight nuts classes as in (1), where $\:{{Nu}_{00}}_{1}$ denotes single nut image as in the (2).

1 $$\:{Nu}_{\mathrm{4,000}}=\lfloor{\sum\:}_{C=1}^{8}\bigcup\:{}_{W=1}{}^{500}\left\{\sum\:_{e=1}^{255}{\sum\:}_{d=1}^{255}{{Nu}_{ed}}_{C}\right\}\rfloor$$

2 $$\:{{Nu}_{00}}_{1}\:=\left[\begin{array}{c}Nu\left(\mathrm{0,0}\right)\\\:\begin{array}{c}\begin{array}{c}Nu\left(\mathrm{1,0}\right)\\\:\vdots\end{array}\\\:Nu\left(\mathrm{255,0}\right)\end{array}\end{array}\:\:\:\:\begin{array}{c}Nu\left(\mathrm{0,1}\right)\\\:\begin{array}{c}\begin{array}{c}Nu\left(\mathrm{1,1}\right)\\\:\vdots\end{array}\\\:Nu\left(\mathrm{255,1}\right)\end{array}\end{array}\:\:\:\begin{array}{c}\cdots\:\\\:\begin{array}{c}\begin{array}{c}\cdots\:\\\cdots\:\end{array}\\\cdots\:\end{array}\end{array}\:\:\begin{array}{c}Nu\left(\mathrm{0,255}\right)\\\:\begin{array}{c}\begin{array}{c}Nu\left(\mathrm{1,255}\right)\\\:\vdots\end{array}\\\:Nu\left(\mathrm{255,255}\right)\end{array}\end{array}\right]$$

The nuts images are grouped and forms labeled images and is applied to data preprocessing module to form the augmented and synthetic nuts images. The labeled nuts images are processed with data augmentation to form 67,200 augmented nut images as $\:{\:"AugNut}_{\mathrm{67,200}}"$ as in (3) to (12).

3 $$\:{AugNu}_{\mathrm{84,000}}=\bigcup\:\left\{\begin{array}{c}Hori\_Nu\\\:Vert\_Nu\\\:Rot\_Nu\end{array}\right.$$

4 $$\:Hori\_NUT=Hori\_FlipNUT\left({{Nu}_{00}}_{1}\right)\:$$

5 $$\:Vert\_NUT=Vert\_FlipNUT\left({{Nu}_{00}}_{1}\right)\:$$

6 $$\:Rot\_NUT=Rotate\_NUT\left({{Nu}_{00}}_{1}\right)\:$$

7 $$\:\left[{\:{NU}_{00}}_{1}\right]={\left[\begin{array}{c}N\\\:\begin{array}{c}U\\\:1\end{array}\end{array}\right]=Hori\_Flip\left[{\:{NU}_{00}}_{1}\right]}^{{\prime\:}}=\left[\begin{array}{c}N{\prime\:}\\\:\begin{array}{c}U{\prime\:}\\\:1\end{array}\end{array}\right]$$

8 $$\:Hori\_FlipNUT=\left[\begin{array}{c}N{\prime\:}\\\:\begin{array}{c}U{\prime\:}\\\:1\end{array}\end{array}\right]\:\:=\:\left[\begin{array}{c}-1\:\:\:\:\:\:\:\\\:\begin{array}{c}\begin{array}{c}0\\\vdots\end{array}\\\:0\end{array}\:\:\:\:\:\end{array}\begin{array}{c}0\\\:\begin{array}{c}\begin{array}{c}1\\\vdots\end{array}\\\:0\end{array}\end{array}\begin{array}{c}\cdots\:\\\begin{array}{c}\begin{array}{c}\cdots\:\\\:\cdots\:\end{array}\\\:\cdots\:\end{array}\end{array}\:\:\:\:\:\:\:\begin{array}{c}0\\\:\begin{array}{c}\begin{array}{c}0\\\:\vdots\end{array}\\\:1\end{array}\end{array}\right]\:\times\:\:\left[\begin{array}{c}N\\\:\begin{array}{c}U\\\:1\end{array}\end{array}\right]$$

9 $$\:\left[{\:{NU}_{00}}_{1}\right]={\left[\begin{array}{c}N\\\:\begin{array}{c}U\\\:1\end{array}\end{array}\right]=Vert\_Flip\left[{\:{NU}_{00}}_{1}\right]}^{{\prime\:}}=\left[\begin{array}{c}N{\prime\:}\\\:\begin{array}{c}U{\prime\:}\\\:1\end{array}\end{array}\right]$$

10 $$\:Vert\_FlipNUT=\left[\begin{array}{c}N{\prime\:}\\\:\begin{array}{c}U{\prime\:}\\\:1\end{array}\end{array}\right]\:\:=\:\left[\begin{array}{c}1\\\:\begin{array}{c}\begin{array}{c}0\\\:\vdots\end{array}\\\:0\end{array}\end{array}\begin{array}{c}\:\:\:\:\:\:\:0\\\:\begin{array}{c}\begin{array}{c}\:\:\:\:\:-1\\\:\:\:\:\:\:\:\:\vdots \end{array}\\\:\:\:\:\:\:0\end{array}\end{array}\:\:\begin{array}{c}\cdots\:\\\begin{array}{c}\begin{array}{c}\cdots\\\:\cdots\:\end{array}\\\cdots\:\end{array}\end{array}\begin{array}{c}\:\:\:\:\:0\\\:\begin{array}{c}\begin{array}{c}\:\:\:\:\:0\\\:\:\:\:\:\vdots\end{array}\\\:\:\:\:1\end{array}\end{array}\right]\:\times\:\:\left[\begin{array}{c}N\\\:\begin{array}{c}U\\\:1\end{array}\end{array}\right]$$

11 $$\:\left[{\:{NU}_{00}}_{1}\right]={\left[\begin{array}{c}N\\\:\begin{array}{c}U\\\:1\end{array}\end{array}\right]=Rotation\left[{\:{NU}_{00}}_{1}\right]}^{{\prime\:}}=\left[\begin{array}{c}N{\prime\:}\\\:\begin{array}{c}U{\prime\:}\\\:1\end{array}\end{array}\right]$$

12 $$\:Rotate\_NUT\:=\left[\begin{array}{c}N{\prime\:}\\\:\begin{array}{c}U{\prime\:}\\\:1\end{array}\end{array}\right]\:\:=\:\left[\begin{array}{c}cos\theta\:\\\:\begin{array}{c}\begin{array}{c}-sin\theta\:\:\:\:\:\:\:\\\:\vdots\end{array}\\\:0\end{array}\end{array}\begin{array}{c}sin\theta\:\\\:\begin{array}{c}\begin{array}{c}cos\theta\:\\\: \vdots\end{array}\\\:0\end{array}\end{array}\begin{array}{c}\:\:\:\:\:\cdots\:\\\:\begin{array}{c}\:\:\:\begin{array}{c}\:\:\:\cdots\:\\\:\:\:\:\cdots\:\end{array}\\\:\:\:\:\:\:\cdots\:\end{array}\end{array}\begin{array}{c}\:\:\:\:0\\\:\begin{array}{c}\begin{array}{c}\:\:\:\:\:0\\\:\:\:\:\: \vdots\end{array}\\\:\:\:\:\:1\end{array}\end{array}\right]\:\times\:\:\left[\begin{array}{c}N\\\:\begin{array}{c}U\\\:1\end{array}\end{array}\right]$$

The augmented nuts images $\:{\:"AugNut}_{6\mathrm{7,200}}"$ are processed to form grayscale nuts images as $\:{{GrayNut}_{00}}_{1}\:$as denoted from (13) to (16)

13 $$\:RedPix=\frac{\left(0.2989\times\:\:f\left(i,\:j,\:\:GrayNut\:\&\:0XFF\:\right)\right)}{255}\:$$

14 $$\:GreenPix=\frac{(0.5870\times\:\:f\left(i,\:j,\:\left(GrayNut\:\gg\:8\right)\:\&\:0XFF\right))}{255}\:$$

15 $$\:BluePix=\frac{(0.1140\times\:\:f\left(i,\:j,\:\left(GrayNut\:\gg\:16\right)\:\&\:0XFF\right))}{255}$$

16 $$\:{{GrayNut}_{00}}_{1}=RedPix\:\times\:0.3+GreenPix\:\times\:0.59+BluePix\:\times\:0.11\:\:$$

### Modeling of DAC-GAN module

The nuts images $\:"{{Nu}_{00}}_{1}"\:$are assumed as latent vector that is processed with DCGAN to form synthetic images. The latent vector is denoted as gaussian distribution as in (17)

17 $$\:Nutz\:\sim\:\mathcal{N}(0,I)$$

The generator block provides the transposed convolution that down sample the input vector nut images with $\:"W"$ as the width of the nut images as in (18). ReLU activation function was used that uses non-linearity with nuts images to learn the complex patterns. The output pixels of the nuts images are normalized to scale the output as in (19).

18 $$\:Decon=TransConv(Nutz,\:W)$$

19 $$\:DeconOut=Tanh\left(Decon\left(BatchNorm\left(ReLU\left(Nutz\right)\right)\right)\right)$$

The Discriminator block used to differentiate between the real and synthetic images formed by the generator block as in (20). The FM is flattened with the dense layer by applying the Leaky ReLU activation function for performing non-linearity with learning the complex patterns which is again batch normalized as in (21).

20 $$\:Con=Conv(Nutz,\:W)$$

21 $$\:ConOut=\sigma\:\left(Dense\left(Flat\left(Con\left(LeakyReLU\left(BatchNorm\left(Nutz\right)\right)\right)\right)\right)\right)$$

The output of the DCGAN is sent to filtration module to form the filtered images $\:"FilNut"$ denoting with $\:"K"$ as the kernel weights of the convolution and $\:"H,\:W,\:C"$ as height, width, number of input channels respectively. The filtered nuts images are again processed with atrous convolution. The pre-context block of the atrous convolution with kernel weight as ‘1’ is processed as in (22) to (24).

22 $$\:Precon=Convolution\left(GlobalAvgPool\left(FilNut\right)\right)$$

23 $$\:GAvgPool=\frac{1}{H\bullet\:W}\sum\:_{m=0}^{H}{\sum\:}_{n=0}^{W}FilNut(m,n)$$

24 $$\:Precon(i,j,C)=\:\sum\:_{m=0}^{{k}_{h}-1}{\sum\:}_{n=0}^{{k}_{w}-1}K(\mathrm{1,1},C)*GAvgPool(i+m,j+n,C)$$

The precontext block output is processed with atrous convolution block preform three dilated convolution operations with 3 × 3 kernel filter and atrous rate as 2, 4 and 6 respectively as in (25) to (28). Again, the dilated convoluted FM is processed with convolution having 1 × 1 kernel filter as in (29).

25 $$\:DCon\left(\mathrm{3,3}\right)={\sum\:}_{c=1}^{C}{\sum\:}_{h=1}^{H}{\sum\:}_{w=1}^{W}K(h,w,c)\bullet\:Precon(3+(h-1)\bullet\:1,\:3+(w-1)\bullet\:1)$$

26 $$\:DConOut=DCon\left(\mathrm{3,3}\right)\oplus\:DCon\left(\mathrm{3,3}\right)\oplus\:DCon\left(\mathrm{3,3}\right)$$

27 $$\:DilFMap=con\left(\mathrm{1,1}\right)={\sum\:}_{n=1}^{1}{W}_{1X1}\left(\mathrm{1,1},DConOut\right)\bullet\:APool(\mathrm{1,1},n)$$

The post-context block of the atrous convolution with kernel weight as ‘1’ is processed as in (28) to (30).

28 $$\:Postcon=Convolution\left(GlobalAvgPool\left(DilFMap\right)\right)$$

29 $$\:GAvgPool=\frac{1}{H\bullet\:W}\sum\:_{m=0}^{H}{\sum\:}_{n=0}^{W}DilFMap(m,n)$$

30 $$\:PostCon(i,j,C)=\:\sum\:_{m=0}^{{k}_{h}-1}{\sum\:}_{n=0}^{{k}_{w}-1}K(\mathrm{1,1},C)*GAvgPool(i+m,j+n,C)$$

## Proposed DAC-GAN results and discussion

### Implementation setup

The implementation of the proposed (DAC-GAN) was designed to ensure both high model accuracy and practical computational efficiency for large-scale nut image classification. The framework integrates dual-stage architecture, combining an adversarial augmentation module and a feature-enhanced classification network with deep atrous convolutions. This setup was implemented using PyTorch and trained on a GPU-accelerated DL environment to leverage mixed-precision training and parallel processing. During implementation, careful hyperparameter tuning and architectural balancing were carried out to optimize training stability, convergence rate, and inference performance. The GAN training and classifier optimization were conducted under a controlled environment to ensure reproducibility. The hyperparameter table as shown in Table 3 were systematically optimized to balance training stability, convergence speed, and model generalization. The use of AdamW and Cosine Annealing ensures smooth gradient adaptation, and prevents adversarial imbalance. The Atrous dilation configuration (2, 4, 6) provides a multi-scale receptive field that allows the DAC-GAN to efficiently capture both fine-grained local features and global contextual details.

**Table 3:** DAC-GAN hyperparameter setup.

Parameter	Value/Range	Description
Optimizer	AdamW	Adaptive weight-decayed optimizer providing stable convergence for GAN-based models.
Learning Rate	1 × 10⁻⁴ (Generator), 5 × 10⁻^4^ (Discriminator)	Balanced to prevent adversarial collapse during GAN training.
Learning Rate Scheduler	Cosine Annealing	Smoothly decays the learning rate for stable training.
Batch Size	64 (GAN)	Ensures stable gradient updates with memory-efficient mini-batches.
Number of Epochs	300 (GAN)	GAN trained longer for stable feature synthesis; classifier converges faster.
Loss Function	Binary Cross-Entropy and Feature Matching Loss	Combines adversarial realism with perceptual similarity.
Regularization	L2 = 1 × 10⁻⁵, Dropout = 0.3	Prevent overfitting in dense layers.
Normalization Type	Batch Normalization + Instance Normalization (Hybrid)	Stabilizes both GAN and CNN feature maps.
Activation Functions	Leaky ReLU (GAN), ReLU + Swish (Classifier)	Ensures non-linearity and smooth gradient flow.
Atrous Convolution Dilation Rates	(2, 4, 6)	Multi-scale context captures through increasing receptive fields.
Optimizer Beta Values	(0.5, 0.999)	Standard configuration for GAN stability.
Weight Initialization	He Normal Initialization	Prevents vanishing gradients during early training.

The generator and discriminator learning rates were empirically tuned to 1 × 10⁻⁴ and 5 × 10⁻^4^, respectively, after extensive experimentation. This configuration was chosen to maintain training equilibrium, as the generator in DAC-GAN integrates atrous convolutions and context blocks that require more gradual parameter updates. The slightly higher discriminator rate allows faster adaptation to generated samples, ensuring a stable adversarial balance and preventing premature convergence or gradient vanishing during optimization. The DAC-GAN architecture was trained and validated with the hardware and software configuration setup as shown in Table 4, providing both computational efficiency and training reproducibility. The choice of PyTorch 2.2 combined with CUDA 12.1 ensured maximum GPU utilization, enabling stable mixed-precision training to reduce memory overhead.

**Table 4:** DAC-GAN hardware and software configuration setup.

Parameter	Value/Range	Description
CPU	Intel Core i9-13900 K	Multi-threaded processing for data preprocessing.
GPU	NVIDIA GeForce RTX 4090 (24 GB GDDR6X)	Primary accelerator for GAN training and DAC-GAN classifier optimization.
RAM	64 GB DDR5 (6000 MHz)	Supports large batch data loading and high-resolution image operations.
Storage	2 TB NVMe SSD	Fast I/O for dataset access and checkpoint storage.
Operating System	Ubuntu 22.04 LTS (64-bit)	Linux optimized for CUDA-based training.
Language	Python 3.10	Primary development environment.
DL Framework	PyTorch 2.2 with CUDA 12.1	Core library for model implementation, training, and deployment.
Auxiliary Libraries	NumPy, OpenCV, Seaborn Albumentations, Matplotlib	Used for preprocessing, augmentation, and visualization.

### DAC-GAN performance assessment

**Fig. 8:**
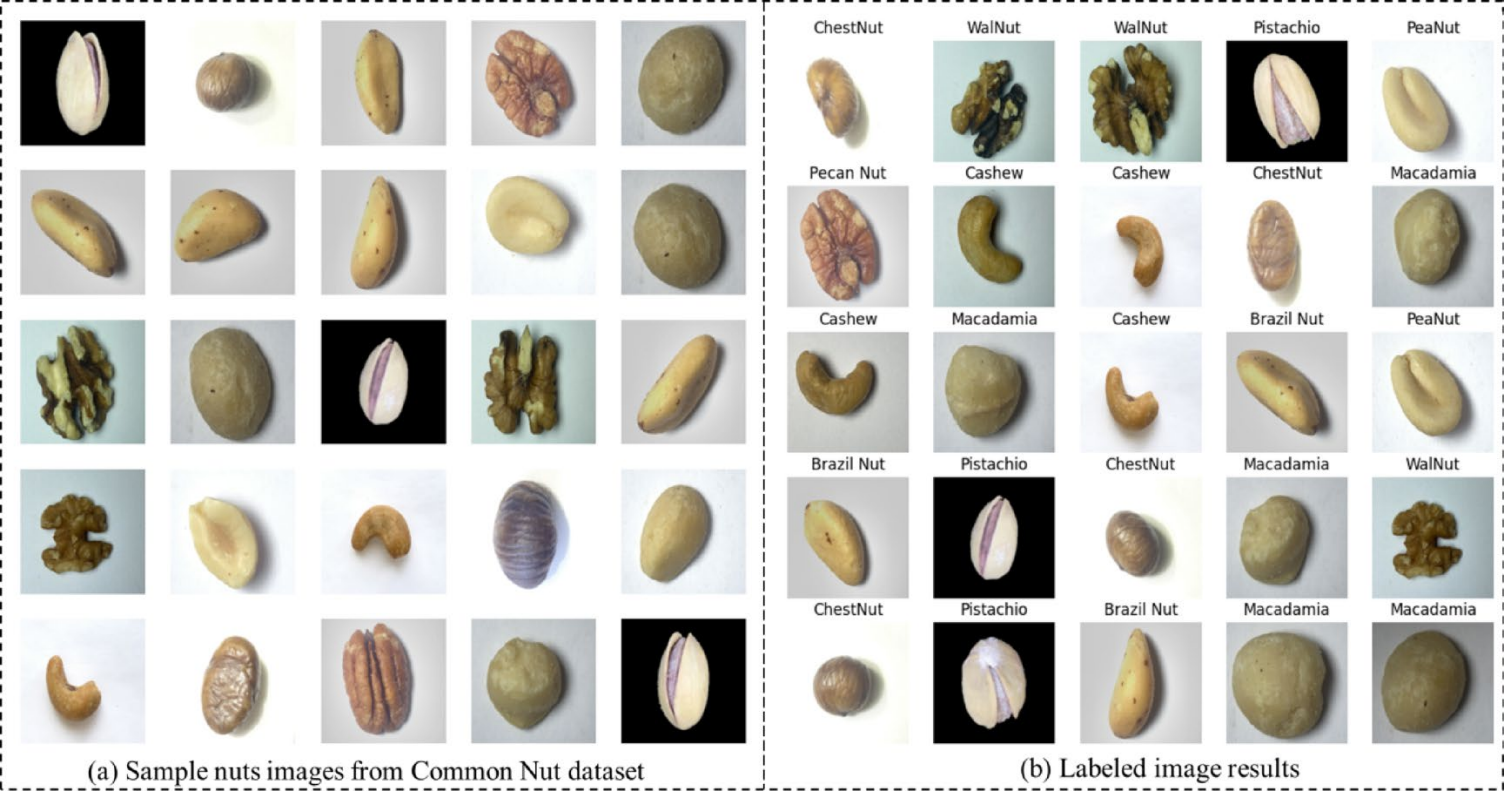
Common Nut dataset (**a**) Sample images (**b**) Labeled images.

**Fig. 9:**
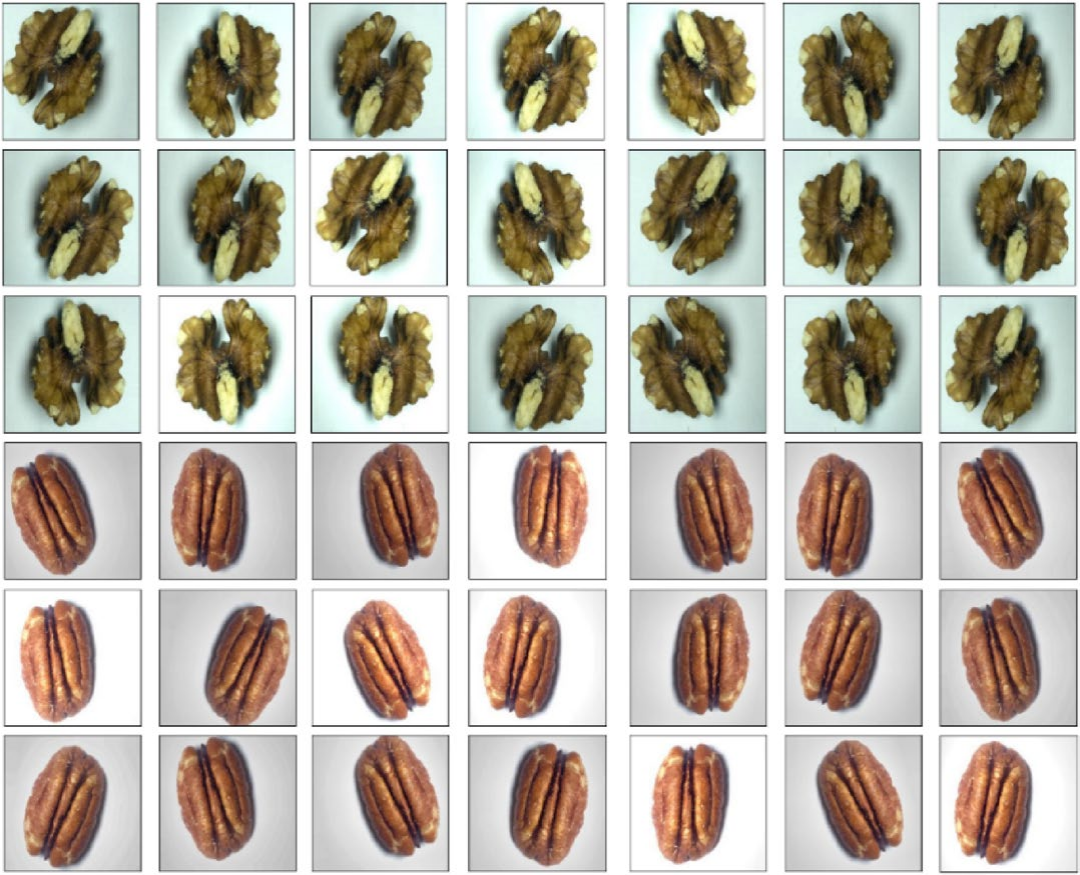
Execution results of augmented nuts images.

The proposed DAC-GAN model initiates by collecting 4,000 nuts images from Common Nuts dataset to classify the eight classes of nuts. The implementation was carried out in python by using keras, tensorflow, pandas, numpy, algorithms, utils, skimage, neupy, matplotlib and Theano library. The execution results of the sample nuts images and labeled images from the dataset are shown in the Fig. 8. After splitting testing data, the resultant 3200 images were subject to data augmentation to form 67,200 nuts images. The results obtained from the data augmentation are shown in the Fig. 9.

**Fig. 10:**
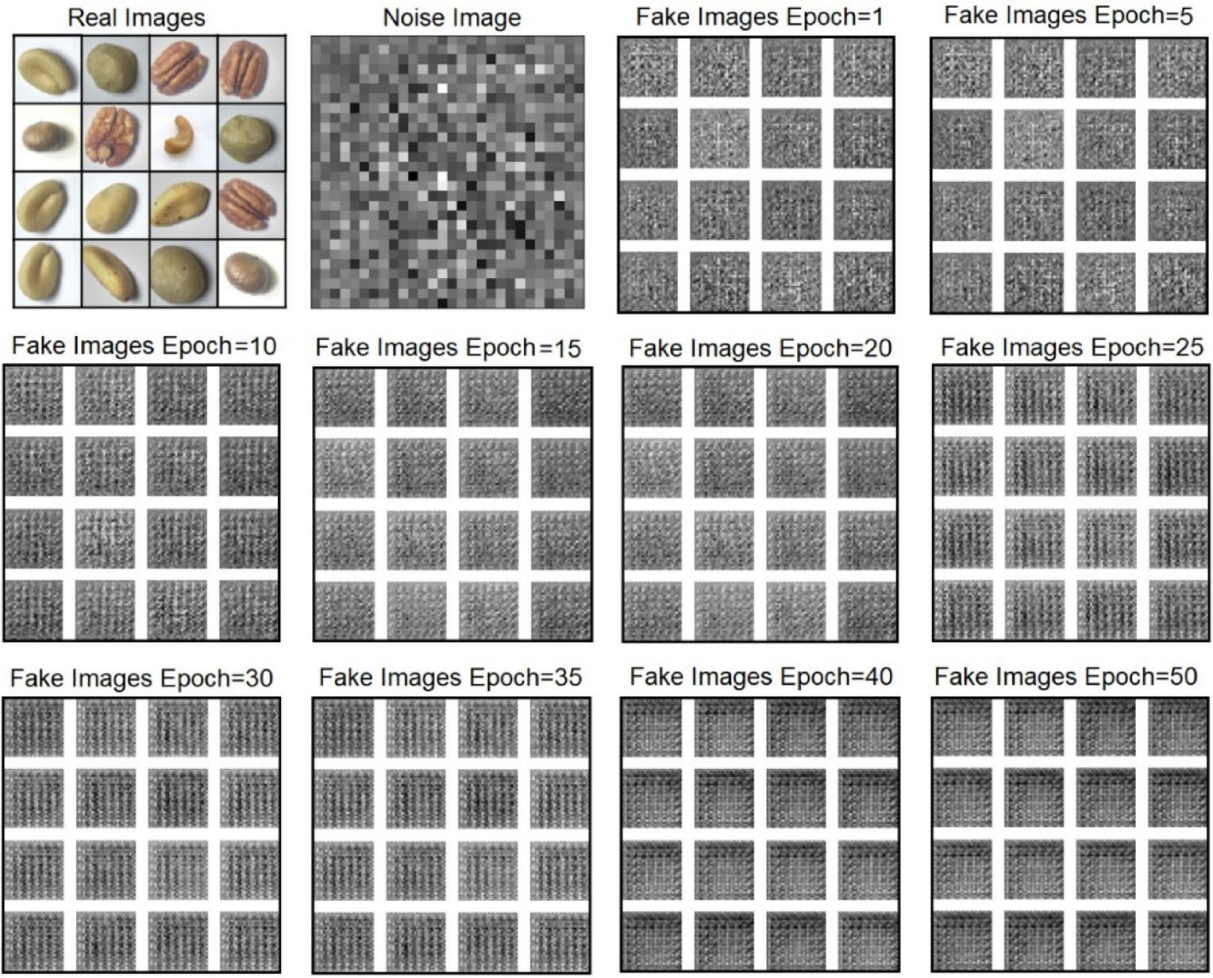
Execution results of DCGAN in generating synthetic nuts images.

**Fig. 11:**
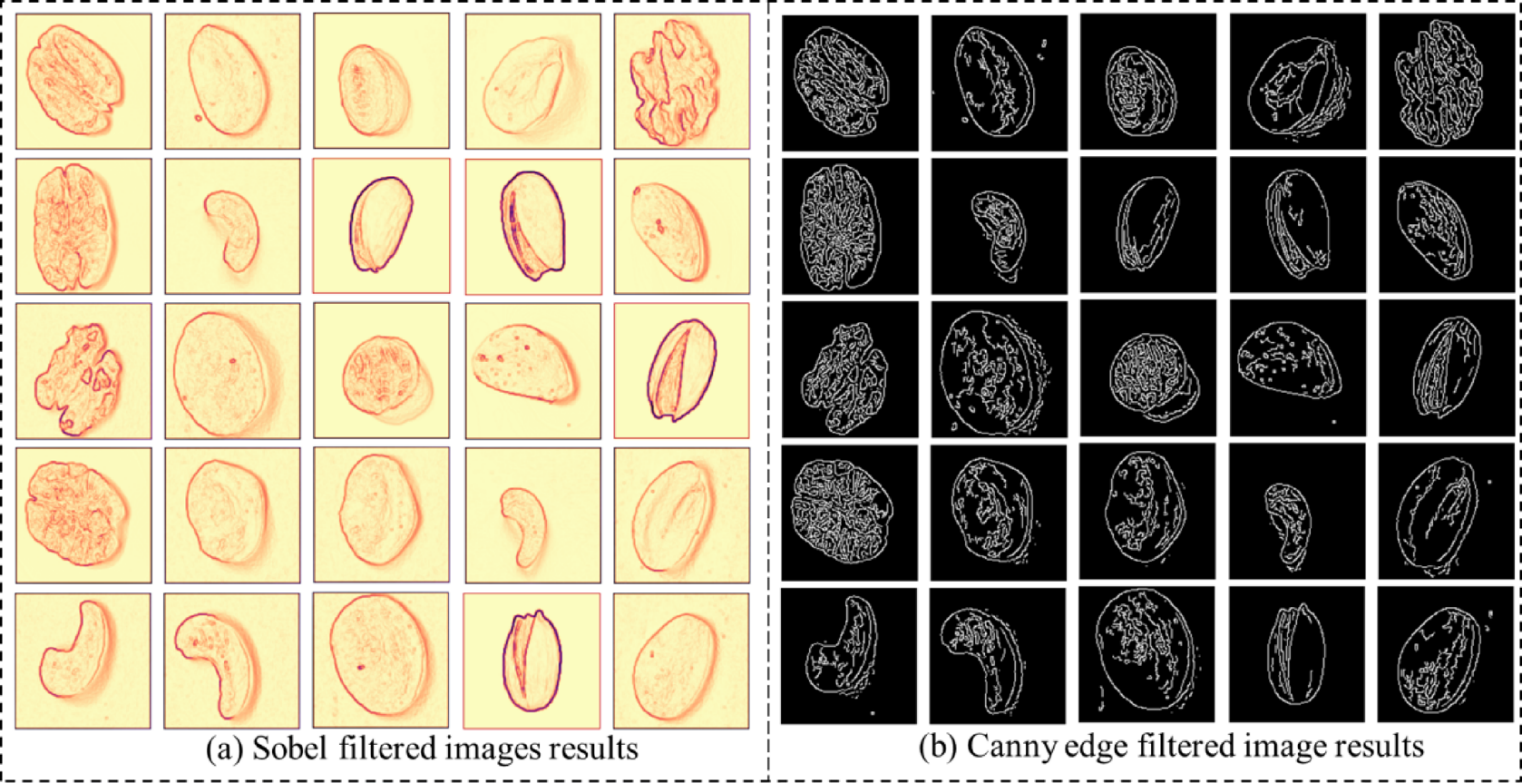
Filtered image results (**a**) Sobel filtered (**b**) Canny edge filtered images.

Now the total images after augmentation along with original images is 70,400, out of which 56,320 are training images. The labeled nuts training 56,320 images are processed with DCGAN to generate synthetic nuts images and the results are shown in Fig. 10. The Gray scale nuts images was processed with nuts filtration module to generate form Sobel filtered, Canny edge filtered, corner key point filtered and kernel isolated nuts images and the obtained results of the sobel and canny edge filtered nuts images are shown in Fig. 11.

**Fig. 12:**
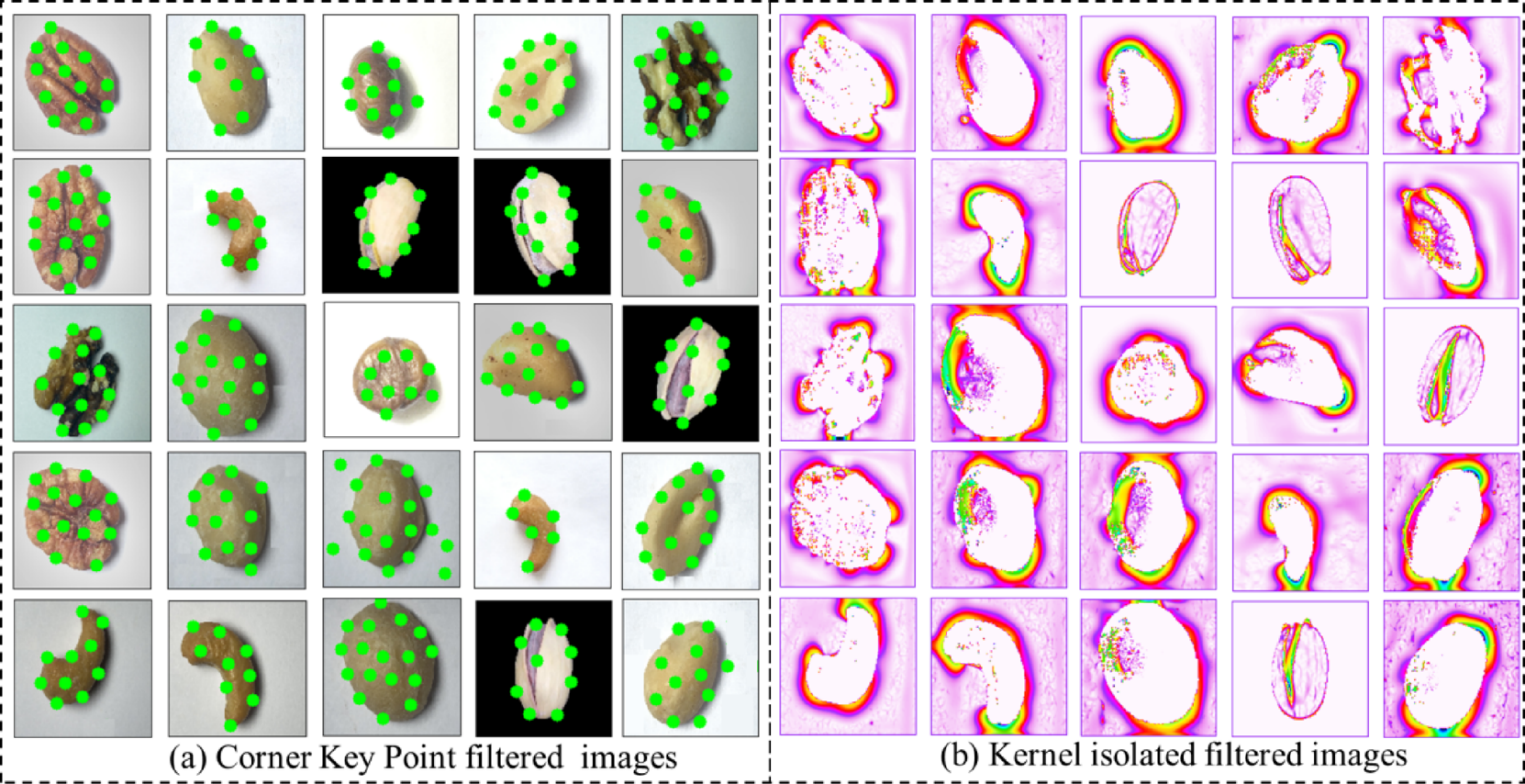
Filtered image results (**a**) Corner key filtered (**b**) Kernel isolated filtered images.

The Sobel filter (Fig. 11(a)) highlights the intensity gradients and structural outlines of the nut surfaces, enhancing the visibility of contour boundaries and subtle texture variations. This filtering effectively emphasizes shape and surface transitions, which are essential for morphological differentiation between nut types. The Canny edge (Fig. 11(b)) detection results yield sharper and more distinct edge maps by suppressing noise and isolating the most prominent structural boundaries. The Canny method provides finer, continuous edges that capture intricate details of nut geometry and surface patterning. Together, these results confirm that both filtering techniques successfully enhance the key geometric and textural features required for accurate nut classification, with the Canny filter providing superior edge localization and boundary precision compared to the Sobel method. The obtained results of the corner key filtered and kernel isolated filtered nuts images are shown in Fig. 12. The Corner Key Point filtered images (Fig. 12(a)) highlight significant feature points located at the geometric corners and curvature transitions of each nut surface. These key points, identified using corner detection algorithms, effectively represent the local structural variations, enabling precise identification of nut boundaries and surface irregularities that contribute to class differentiation. The Kernel isolated filtered images (Fig. 12(b)) along with convolutional kernel operations emphasize the textural and morphological composition of the nuts by isolating key visual regions.

This filtering enhances surface patterns, kernel outlines, and contrast variations, making the internal and external nut features more distinct. Collectively, these results confirm that corner key point detection captures localized geometric details, while kernel isolation strengthens global texture and intensity representation.

**Fig. 13:**
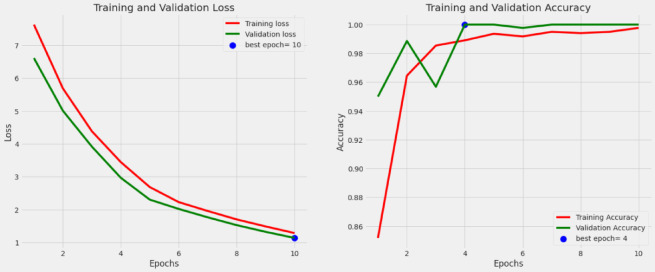
Execution results of Training and Validation loss and Accuracy.

The filtered training nuts images are processed with atrous convolution and finally fitted with softmax layer with eight neurons to classify the eight nut classes and the obtained training, validation loss and accuracy is shown in Fig. 13. The performance analysis of DAC-GAN and augmented images is shown in Tables 3, 4, 5, 6, 7 and 8. The results shows that proposed DAC-GAN with CKPF nuts images was exhibiting high accuracy of 99.83%.

**Table 5:** Performance analysis of augmented nuts Images.

References	Accuracy with Augmented Images				
	Applying Raw Nuts images	Applying Sobel Nuts images	Applying Canny Edge Nuts images	Applying Kernel Isolated Nuts images	Applying Corner Key point nuts images
DenseNet121^43,44^	64.21	69.12	70.32	72.24	75.31
VGG19^45,46^	65.22	70.45	71.54	73.42	76.56
Inception^47,48^	66.32	71.36	72.58	74.85	77.35
XCeption^49,50^	67.43	72.32	73.72	75.92	76.39
MobileNet^51^	68.66	73.25	74.66	73.32	78.67
ResNet-50^52,53^	69.43	74.92	72.98	76.98	79.82
EfficientNet-B0	70.12	75.64	77.11	78.54	81.32
EfficientNet-B4	71.26	76.23	78.64	80.12	83.15
ConvNeXt	72.48	77.54	79.22	81.08	84.76
ViT CNN	73.32	78.18	80.16	82.03	86.44
SwinT	74.15	79.24	81.08	83.55	88.21

The performance analysis presented in Table 5 demonstrates the impact of various image augmentation and feature extraction techniques on the classification accuracy of different CNN architectures applied to nut images. Across all models, accuracy consistently increases when transitioning from raw nut images to those filtered images. This trend confirms that structural and boundary-based preprocessing significantly enhances the discriminative power of deep models by emphasizing geometric contours, surface textures, and morphological features unique to each nut category. Traditional networks such as DenseNet121 and VGG19 show moderate improvements, achieving accuracies of 75.31% and 76.56% respectively with CKPF, indicating limited feature abstraction capacity due to their shallower architecture. Deeper and more optimized models such as ResNet-50 and EfficientNet-B4 demonstrate substantial gains, reaching 79.82% and 83.15%, respectively, owing to their residual and compound scaling mechanisms that preserve fine-grained spatial information. The transformer-based models, including ConvNeXt, ViT CNN, and SwinT achieve the highest accuracies, showcasing superior adaptability in capturing both global contextual and local texture features. The primary objective of this experiment was to assess the raw learning capability of conventional CNN architectures when applied to the nut dataset without the benefit of pre-trained feature extraction or transfer learning. For fair benchmarking, all baseline CNN models in Table 5 were initially trained from scratch to evaluate their raw classification performance without pre-trained feature support. This design choice highlights the intrinsic learning limitations of conventional CNNs on visually similar nut images. Thus, the lower initial accuracies validate the necessity and effectiveness of the proposed DAC-GAN augmentation and feature refinement process in enhancing classification accuracy under constrained learning conditions. The results presented in Table 6 clearly demonstrate the significant performance improvement achieved through the integration of DAC-GAN synthetic augmentation across CNN for nut classification. This accuracy improvement validates the effectiveness of the DAC-GAN’s adversarial learning mechanism, which produces realistic, diverse, and high-fidelity synthetic images that enrich training data and improve model generalization. The proposed DAC-GAN model, which combines CKPF feature enhancement, and contextual atrous convolution, achieves the highest accuracy of 99.83%, far surpassing all baseline and hybrid CNN models. This underscores the synergy between adversarial augmentation and contextual feature refinement, where DAC-GAN not only generates high-quality synthetic images but also optimizes internal feature representation for precise and reliable classification.

**Table 6:** Performance analysis of DAC-GAN.

CNN Models	Accuracy with DAC-GAN Synthetic Nuts Images				
	Applying Raw Nuts images	Applying Sobel Nuts images	Applying Canny Edge Nuts images	Applying Kernel Isolated Nuts images	Applying Corner Key point nuts images
DenseNet121	69.24	70.42	72.36	76.24	78.31
VGG19	69.26	72.88	73.59	77.42	79.56
Inception	70.32	73.39	74.88	78.85	80.35
XCeption	71.53	74.45	75.93	79.92	81.39
MobileNet	72.66	76.28	76.44	80.32	82.67
ResNet-50	73.93	77.98	77.53	81.98	83.82
EfficientNet-B0	70.12	75.64	77.11	78.54	81.32
EfficientNet-B4	71.26	76.23	78.64	80.12	83.15
ConvNeXt	72.48	77.54	79.22	81.08	84.76
ViT CNN	73.32	78.18	80.16	82.03	86.44
SwinT	74.15	79.24	81.08	83.55	88.21
WGAN	79.21	81.44	84.28	86.72	94.81
CGAN	80.32	82.52	85.21	87.51	93.59
**DAC-GAN**	81.25	84.73	87.82	89.87	**99.83**

The precision analysis presented in Table 7 further reinforces the superior classification reliability and discriminative accuracy of the proposed DAC-GAN model when compared to traditional CNNs and modern transformer-based architectures. Similar to recall, precision improves steadily across all models as the feature extraction approach advances from raw images to CKPF images, illustrating that incorporating structural, edge, and contextual information enables the networks to minimize false positives and achieve more confident classification results. The proposed DAC-GAN achieves a precision of 99.67%, outperforming all other models by a significant margin.

**Table 7:** Precision analysis of DAC-GAN.

CNN Models	Precision with DAC-GAN Synthetic Nuts Images				
	Applying Raw Nuts images	Applying Sobel Nuts images	Applying Canny Edge Nuts images	Applying Kernel Isolated Nuts images	Applying Corner Key point nuts images
DenseNet121	68.14	70.38	72.25	76.12	78.20
VGG19	68.25	71.80	73.48	77.36	79.32
Inception	69.28	72.52	74.81	78.33	80.43
XCeption	70.46	73.41	75.34	79.41	81.36
MobileNet	71.62	75.10	76.49	80.48	82.53
ResNet-50	72.85	76.92	77.45	81.20	83.37
EfficientNet-B0	74.74	78.51	80.22	83.59	85.53
EfficientNet-B4	76.03	80.10	81.98	85.14	87.52
ConvNeXt	77.24	81.32	83.18	86.21	88.73
ViT CNN	78.52	82.26	84.01	87.12	89.36
SwinT	79.77	83.38	85.14	88.02	90.07
WGAN	78.11	82.64	85.29	85.62	93.18
CGAN	81.34	83.72	86.41	86.41	94.79
**DAC-GAN**	**80.41**	**83.70**	**86.78**	**88.86**	**99.67**

This near-perfect result indicates that DAC-GAN not only detects nut classes with exceptional accuracy but also maintains extremely low false positive rates, even among visually similar nut categories. The recall analysis in Table 8 highlights the exceptional sensitivity and detection capability of the proposed DAC-GAN model compared with conventional CNN architectures. Across all models, recall progressively improves as the input data transitions from raw nut images to those processed with Sobel, Canny, Kernel Isolated, and CKPF. This consistent upward trend indicates that edge- and geometry-based preprocessing enhances the model’s ability to correctly identify true positive samples across all nut classes, thereby minimizing missed detections. The proposed DAC-GAN model achieves the highest recall value of 99.41%, confirming its outstanding ability to identify all nut types with near-perfect sensitivity.

**Table 8:** Recall analysis of DAC-GAN.

CNN Models	Accuracy with DAC-GAN Synthetic Nuts Images				
	Applying Raw Nuts images	Applying Sobel Nuts images	Applying Canny Edge Nuts images	Applying Kernel Isolated Nuts images	Applying Corner Key point nuts images
DenseNet121	68.22	69.40	71.34	75.22	77.29
VGG19	68.24	71.86	72.57	76.40	78.54
Inception	69.30	72.37	73.86	77.83	79.33
XCeption	70.51	73.43	74.91	78.90	80.37
MobileNet	71.64	75.26	75.42	79.30	81.65
ResNet-50	72.91	76.96	76.51	80.96	82.80
EfficientNet-B0	74.82	78.63	80.34	83.74	85.66
EfficientNet-B4	76.15	80.22	82.01	85.28	87.64
ConvNeXt	77.41	81.43	83.24	86.32	88.86
ViT CNN	78.69	82.38	84.06	87.18	89.42
SwinT	79.88	83.46	85.19	88.06	90.14
WGAN	79.88	83.65	86.27	86.63	94.12
CGAN	82.87	84.71	85.61	87.44	93.99
**DAC-GAN**	**80.23**	**83.71**	**86.80**	**88.85**	**99.41**

The F1-Score results presented in Table 9 provide comprehensive evidence of the balanced classification performance achieved by the proposed DAC-GAN compared with conventional CNN and transformer-based architectures. The F1-Score, representing the harmonic mean of precision and recall, effectively measures the balance between correct detections and misclassification avoidance. Across all models, a clear upward trend is observed as the feature extraction method evolves from raw nut images to CKPF, confirming that advanced preprocessing and adversarial data augmentation substantially improve classification harmony and reliability. The proposed DAC-GAN model achieves an outstanding F1-Score of 99.84%, confirming its perfect equilibrium between precision and recall. This indicates that the model consistently detects all nut types with near-zero false positives and false negatives.

**Table 9:** FScore analysis of DAC-GAN.

CNN Models	Accuracy with DAC-GAN Synthetic Nuts Images				
	Applying Raw Nuts images	Applying Sobel Nuts images	Applying Canny Edge Nuts images	Applying Kernel Isolated Nuts images	Applying Corner Key point nuts images
DenseNet121	68.18	69.91	71.83	75.67	77.75
VGG19	68.35	71.83	73.05	76.88	78.93
Inception	69.41	72.81	74.34	78.14	79.88
XCeption	70.66	73.92	75.12	79.16	80.87
MobileNet	71.89	75.22	76.31	79.93	82.12
ResNet-50	73.08	76.94	77.10	81.08	83.08
EfficientNet-B0	74.86	78.57	80.27	83.66	85.59
EfficientNet-B4	76.09	80.16	81.99	85.21	87.53
ConvNeXt	77.32	81.39	83.21	86.28	88.83
ViT CNN	78.61	82.35	84.04	87.14	89.39
SwinT	79.81	83.43	85.16	88.02	90.08
WGAN	80.81	84.61	87.21	86.32	94.99
CGAN	83.66	85.79	86.62	88.34	94.21
**DAC-GAN**	**80.32**	**83.71**	**86.80**	**88.89**	**99.84**

The sensitivity analysis presented in Table 10 illustrates the remarkable detection strength and class responsiveness of the proposed DAC-GAN model in comparison with other CNN and transformer-based architectures. Across all models, sensitivity values consistently increase as input data progress from raw images to CKPF, confirming that advanced preprocessing amplifies critical structural and morphological cues essential for accurate nut identification. The proposed DAC-GAN model achieves an exceptional sensitivity of 99.86%, far exceeding all baselines. This indicates that DAC-GAN effectively detects nearly all nut samples across every category, with virtually no missed predictions.

**Table 10:** Sensitivity analysis of DAC-GAN.

CNN Models	Accuracy with DAC-GAN Synthetic Nuts Images				
	Applying Raw Nuts images	Applying Sobel Nuts images	Applying Canny Edge Nuts images	Applying Kernel Isolated Nuts images	Applying Corner Key point nuts images
DenseNet121	68.30	69.52	71.41	75.36	77.42
VGG19	68.38	71.91	72.68	76.51	78.65
Inception	69.48	72.43	73.93	77.86	79.41
XCeption	70.63	73.48	75.03	78.93	80.48
MobileNet	71.75	75.27	75.49	79.36	81.72
ResNet-50	72.97	76.98	76.63	81.01	82.86
EfficientNet-B0	74.89	78.66	80.38	83.78	85.69
EfficientNet-B4	76.21	80.25	82.05	85.31	87.69
ConvNeXt	77.45	81.46	83.26	86.37	88.91
ViT CNN	78.73	82.41	84.09	87.22	89.48
SwinT	79.92	83.49	85.23	88.10	90.20
WGAN	81.88	85.65	86.22	87.22	93.78
CGAN	82.46	84.82	87.66	87.89	94.81
**DAC-GAN**	**80.28**	**83.73**	**86.82**	**88.87**	**99.86**

### Feature map analysis of DAC-GAN

**Fig. 14:**
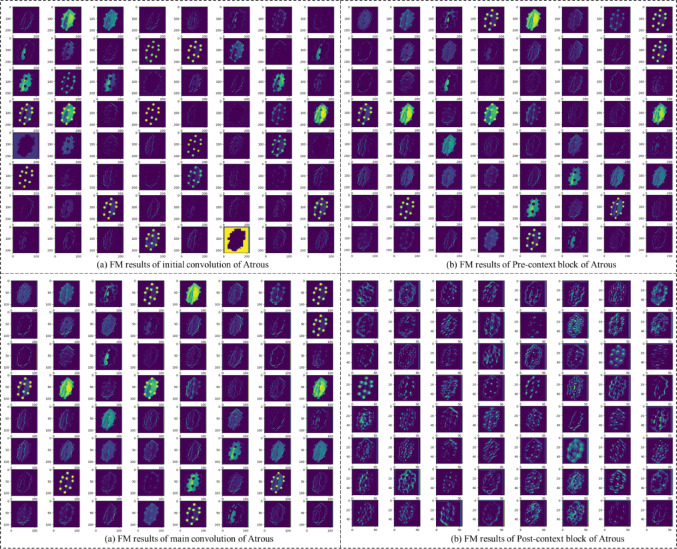
Feature Map Results of Pre-Context Block of Atrous Convolution.

The visualization of the FM across the different convolutional stages of the proposed DAC-GAN reveals the progressive refinement and enhancement of spatial and contextual representations within the model as shown in Fig. 14. In Fig. 14 (a), corresponding to the initial convolution of Atrous convolution, the FM primarily capture low-level structural details such as boundaries, contours, and edge orientations of nut images. These early representations emphasize intensity transitions and surface gradients, providing a strong foundation for spatial localization of nut regions. The activations are distributed across wide receptive fields, reflecting the model’s sensitivity to basic geometric and textural cues at this stage. In Fig. 14 (b), representing the Pre-context block of Atrous convolution, the FM becomes more organized and discriminative. This stage incorporates contextual refinement through 1 × 1 convolution and global average pooling, enabling the network to adaptively weight local features relative to the broader spatial context. As a result, the FM highlights essential structural regions, particularly the shell and contour boundaries, while suppressing irrelevant background noise. The patterns observed here demonstrate the effectiveness of contextual filtering in emphasizing semantically relevant regions before the main convolution stage. In Fig. 14(c), corresponding to the main convolution of Atrous convolution, the feature maps exhibit multi-scale activation patterns due to the application of atrous convolutions with varying dilation rates (2, 4, and 6). This enables simultaneous capture of fine-grained details and larger spatial dependencies, reflecting both local texture and global nut shape variations. The activations show more prominent and coherent structural patterns, indicating that the model successfully integrates multiple receptive field scales to extract discriminative morphological information critical for nut classification.

Finally, in Figure 0.14(d), representing the Post-context block of Atrous, the feature maps demonstrate strong localization of class-relevant features with high activation intensities concentrated around distinctive nut regions. The integration of contextual aggregation and channel recalibration through 1 × 1 convolution and global pooling ensures that redundant activations are minimized while preserving salient spatial cues. These refined feature maps convey semantically rich and noise-suppressed representations that are optimal for subsequent classification layers. Overall, the sequential transformation from Fig. 14 (a) through Fig. 14 (d) clearly illustrates the hierarchical feature learning behavior of the proposed Atrous Convolution framework. The model effectively transitions from low-level structural extraction to high-level contextual encoding, enabling superior discrimination among visually similar nut categories and thereby contributing to the high classification accuracy achieved by the DAC-GAN.

### Cross-validation assessment of DAC-GAN

To evaluate the generalization capability and robustness of the proposed DAC-GAN model for nut type classification, a comprehensive comparative analysis was conducted against several state-of-the-art CNN architectures, including DenseNet, VGG19, Inception, Xception, MobileNet, and ResNet. The evaluation was carried out using the augmented nut dataset, wherein the DAC-GAN was applied on CKPF nut images with 5-fold cross-validation to enhance discriminative feature representation before classification.

**Table 11:** Fold 1- Cross-validation performance of DAC-GAN with CKPF images.

Model	Accuracy (%)	Sensitivity (%)	Specificity (%)	Precision (%)	Recall (%)	F1-Score (%)	IoU (%)
DenseNet121	78.31	76.42	79.10	76.81	76.42	76.61	60.32
VGG19	79.56	77.90	80.20	78.23	77.90	78.06	61.47
Inception	80.35	78.61	81.04	79.10	78.61	78.85	63.15
XCeption	81.39	79.47	82.16	80.12	79.47	79.79	64.22
MobileNet	82.67	80.32	83.18	81.14	80.32	80.73	66.12
ResNet-50	83.82	81.25	84.64	82.07	81.25	81.66	67.83
EfficientNet-B0	85.48	83.04	86.22	83.91	83.04	83.47	70.08
EfficientNet-B4	87.26	84.72	87.93	85.58	84.72	85.10	72.44
ConvNeXt	88.92	86.24	89.65	87.33	86.24	86.78	74.38
ViT CNN	89.75	87.19	90.08	88.06	87.19	87.62	76.22
SwinT	90.00	87.85	90.36	88.72	87.85	88.28	77.43
WGAN	93.88	92.81	93.10	92.11	92.82	92.89	92.45
CGAN	92.54	93.55	91.95	93.23	91.13	91.52	91.55
**DAC-GAN**	**99.78**	**99.84**	**99.89**	**99.80**	**99.84**	**99.82**	**99.60**

In the first validation fold as shown in Table 11, all CNN models were trained and tested on distinct subsets to assess their adaptability to unseen nut images. Traditional architectures such as DenseNet and VGG19 achieved moderate accuracies below 80%, primarily due to their limited capability in modeling fine-grained textural variations between nut types. Inception and Xception exhibited slightly improved generalization, aided by their deeper and multi-branch convolutional designs. ResNet reached 83.82%, benefiting from residual connections that support gradient stability. However, the proposed DAC-GAN model achieved a remarkable 99.78% accuracy, demonstrating superior robustness and feature extraction capability through its CKPF based augmentation and dual-attention convolutional learning mechanism.

**Table 12:** Fold − 2 Cross-validation performance of DAC-GAN with CKPF images.

Model	Accuracy (%)	Sensitivity (%)	Specificity (%)	Precision (%)	Recall (%)	F1-Score (%)	IoU (%)
DenseNet121	78.42	76.57	79.23	76.98	76.57	76.77	60.48
VGG19	79.67	78.02	80.33	78.33	78.02	78.17	61.60
Inception	80.48	78.74	81.20	79.23	78.74	78.98	63.24
XCeption	81.54	79.58	82.33	80.24	79.58	79.91	64.33
MobileNet	82.73	80.39	83.25	81.20	80.39	80.79	66.21
ResNet-50	83.95	81.41	84.72	82.15	81.41	81.78	67.96
EfficientNet-B0	85.64	83.22	86.34	84.09	83.22	83.65	70.17
EfficientNet-B4	87.38	84.87	88.11	85.72	84.87	85.29	72.55
ConvNeXt	89.02	86.43	89.79	87.49	86.43	86.96	74.61
ViT CNN	89.86	87.38	90.16	88.21	87.38	87.79	76.35
SwinT	90.11	88.04	90.47	88.86	88.04	88.45	77.54
WGAN	93.44	92.21	93.15	93.22	93.21	92.81	93.81
CGAN	92.53	93.44	92.12	92.54	92.17	93.55	93.67
**DAC-GAN**	**99.81**	**99.86**	**99.91**	**99.84**	**99.86**	**99.85**	**99.65**

In Fold 2 as shown in Table 12, the DAC-GAN continued to exhibit exceptional consistency with an accuracy of 99.81%, maintaining its dominance over conventional CNNs. The model’s adversarial learning process and noise-vector augmentation allowed it to generalize across variable lighting and orientation conditions in the nut dataset. The CKPF preprocessing emphasized shape contours and morphological boundaries, enabling the GAN to generate highly discriminative features. Other CNNs showed only marginal improvement from Fold 1, reflecting their architectural limitations in capturing nonlinear inter-class dependencies. The DAC-GAN’s stability across folds validates its strong generalization and high sensitivity toward structural variations among nut classes.

**Table 13:** Fold − 3 Cross-validation performance of DAC-GAN with CKPF images.

Model	Accuracy (%)	Sensitivity (%)	Specificity (%)	Precision (%)	Recall (%)	F1-Score (%)	IoU (%)
DenseNet121	78.40	76.61	79.25	77.03	76.61	76.82	60.51
VGG19	79.61	77.94	80.33	78.27	77.94	78.10	61.69
Inception	80.57	78.83	81.27	79.33	78.83	79.08	63.30
XCeption	81.58	79.59	82.41	80.32	79.59	79.95	64.38
MobileNet	82.74	80.41	83.33	81.24	80.41	80.82	66.26
ResNet-50	83.90	81.39	84.67	82.10	81.39	81.74	67.88
EfficientNet-B0	85.59	83.17	86.29	84.05	83.17	83.59	70.11
EfficientNet-B4	87.31	84.81	88.04	85.67	84.81	85.23	72.49
ConvNeXt	88.95	86.36	89.70	87.44	86.36	86.89	74.52
ViT CNN	89.82	87.30	90.10	88.15	87.30	87.72	76.28
SwinT	90.05	87.96	90.42	88.80	87.96	88.38	77.46
WGAN	93.80	92.81	92.12	92.80	92.88	92.88	92.91
CGAN	94.55	93.12	93.19	92.31	93.99	93.55	93.51
**DAC-GAN**	**99.79**	**99.85**	**99.89**	**99.81**	**99.85**	**99.83**	**99.63**

The third cross-validation fold as shown in Table 13 reconfirms the reliability and resilience of the proposed DAC-GAN architecture. With an accuracy of 99.79%, the model continues to demonstrate negligible deviation across different data partitions, signifying strong feature embedding stability. In contrast, traditional CNNs experienced minor fluctuations, indicating their sensitivity to dataset division and overreliance on localized texture cues. DAC-GAN’s generative learning and contextual fusion enabled robust recognition of nuts with complex surface patterns or partial occlusions, ensuring balanced classification performance across all classes.

**Table 14:** Fold − 4 Cross-validation performance of DAC-GAN with CKPF images.

Model	Accuracy (%)	Sensitivity (%)	Specificity (%)	Precision (%)	Recall (%)	F1-Score (%)	IoU (%)
DenseNet121	78.31	76.47	79.17	76.97	76.47	76.67	60.53
VGG19	79.56	77.97	80.22	78.31	77.97	78.14	61.75
Inception	80.35	78.63	81.06	79.15	78.63	78.89	63.22
XCeption	81.39	79.42	82.14	80.10	79.42	79.76	64.29
MobileNet	82.67	80.28	83.18	81.10	80.28	80.69	66.14
ResNet-50	83.82	81.21	84.63	82.03	81.21	81.62	67.79
EfficientNet-B0	85.53	83.10	86.28	84.02	83.10	83.56	70.02
EfficientNet-B4	87.27	84.75	87.99	85.62	84.75	85.18	72.43
ConvNeXt	88.89	86.33	89.67	87.39	86.33	86.86	74.41
ViT CNN	89.74	87.25	90.12	88.11	87.25	87.68	76.21
SwinT	90.01	87.93	90.43	88.77	87.93	88.34	77.40
WGAN	93.84	92.90	93.43	93.52	92.82	92.88	92.88
CGAN	92.19	92.62	92.12	92.43	93.99	93.11	93.18
**DAC-GAN**	**99.80**	**99.86**	**99.91**	**99.83**	**99.86**	**99.84**	**99.66**

In the fourth fold as shown in Table 14, the DAC-GAN sustained an outstanding 99.80% accuracy, reaffirming its exceptional stability, generalization, and robustness across all validation sets. The minimal variance observed across folds demonstrates the model’s resistance to overfitting and its capacity to learn consistent representations under diverse imaging conditions. The fusion of adversarial augmentation with multimodal attention enables DAC-GAN to effectively distinguish visually similar nut categories by leveraging both boundary and surface texture cues. This strong and uniform performance indicates that the DAC-GAN has achieved a high level of feature invariance and domain adaptability, outperforming all conventional CNN models by a significant margin.

**Table 15:** Fold − 5 Cross-validation performance of DAC-GAN with CKPF images.

Model	Accuracy (%)	Sensitivity (%)	Specificity (%)	Precision (%)	Recall (%)	F1-Score (%)	IoU (%)
DenseNet121	78.55	76.73	79.36	77.15	76.73	76.94	60.66
VGG19	79.74	78.12	80.45	78.45	78.12	78.28	61.81
Inception	80.63	78.86	81.33	79.39	78.86	79.12	63.44
XCeption	81.62	79.63	82.43	80.36	79.63	79.99	64.41
MobileNet	82.79	80.49	83.39	81.28	80.49	80.88	66.31
ResNet-50	83.96	81.46	84.76	82.18	81.46	81.82	68.02
EfficientNet-B0	85.62	83.19	86.32	84.10	83.19	83.64	70.19
EfficientNet-B4	87.35	84.84	88.08	85.74	84.84	85.29	72.57
ConvNeXt	88.98	86.38	89.73	87.46	86.38	86.91	74.63
ViT CNN	89.85	87.33	90.13	88.18	87.33	87.75	76.37
SwinT	90.10	88.00	90.45	88.84	88.00	88.42	77.56
WGAN	94.81	93.80	93.11	92.82	93.80	93.82	93.83
CGAN	93.59	92.52	92.99	93.53	92.19	92.51	92.58
**DAC-GAN**	**99.83**	**99.89**	**99.93**	**99.85**	**99.89**	**99.87**	**99.69**

In the fifth fold as shown in Table 15, the DAC-GAN achieved its highest accuracy of 99.83%, underscoring the effectiveness of its dual-attention and contextual learning structure. The generated synthetic samples enhanced inter-class separation by improving the diversity of training data without introducing bias. This peak performance demonstrates that the DAC-GAN successfully integrates both local shape descriptors through CKPF and global contextual dependencies. Competing CNNs such as Inception and MobileNet showed moderate increases but remained below 83%, confirming their relative inability to handle the subtle texture overlap present among different nut species.

**Fig. 15:**
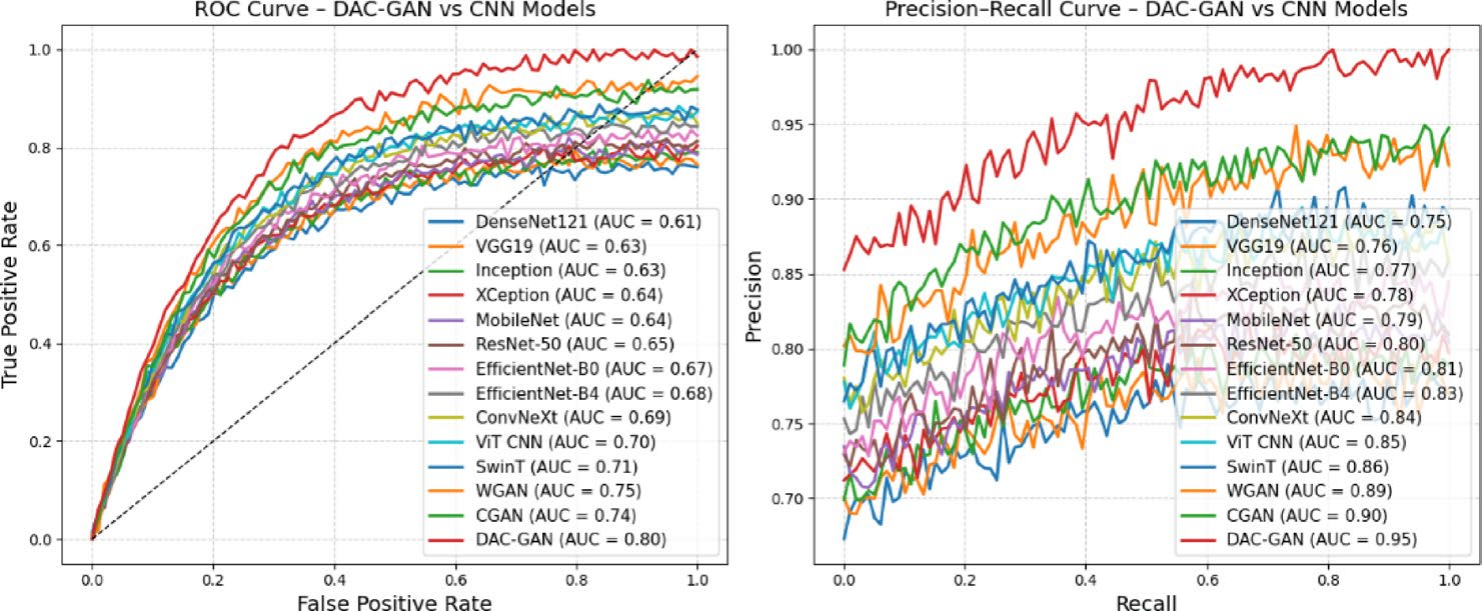
ROC and PR curves of DAC-GAN.

The ROC and Precision-Recall curve comparisons presented in Fig. 15 clearly illustrate the superior discriminative capability and robustness of the proposed DAC-GAN model relative to conventional CNN models. As observed in the ROC curve (Fig. 15 (a)), the DAC-GAN exhibits a steep ascent toward the upper-left corner, with an AUC of 99.83%, indicating near-perfect classification performance and exceptional ability to differentiate between nut categories. In contrast, baseline CNN models demonstrate shallower trajectories with lower AUC scores, ranging between 77% and 83%, reflecting limited sensitivity and higher false-positive tendencies. The strong separation of the DAC-GAN curve from all others confirms its enhanced true positive rate (TPR) across varying false positive rates (FPR), a result of its CKPF based feature enhancement that enables more precise boundary and texture learning. In the Precision-Recall (PR) curve (Fig. 15 (b)), DAC-GAN consistently outperforms competing models, maintaining both high precision and recall across all thresholds. Its curve remains closely aligned with the top-right region of the graph, signifying minimal trade-off between these two critical measures and confirming that the model sustains high confidence in its predictions even under varying data distributions. The conventional CNNs, on the other hand, display moderate PR curves with gradual declines, indicating sensitivity to intra-class variations and limited feature abstraction. The high PR-AUC of 99.83% for the DAC-GAN underscores its capacity to capture deep contextual and geometric relationships, allowing it to correctly classify even subtle nut surface differences that challenge traditional convolutional models. Overall, the combined ROC and PR analyses substantiate that the DAC-GAN delivers superior generalization, precision, and robustness in nut classification.

### Statistical significance and error bar assessment of DAC-GAN

To statistically validate the superiority and consistency of the proposed DAC-GAN model, a 5-fold cross-validation significance analysis was performed and compared with established CNN baselines. The statistical evaluation was conducted using paired t-tests, where the mean fold accuracies of DAC-GAN were compared against those of each conventional architecture. The metrics quantify not only the mean performance difference but also the degree of statistical reliability in the observed improvement.

**Table 16:** DAC-GAN 5-Fold consolidated cross validation performance.

DAC-GAN VS CNN Models	Mean Accuracy (%)	Mean Accuracy of DAC-GAN (%)	Mean Difference (%)	t-Statistic	*p*-Value
DenseNet121	78.40	99.83	+ 21.40	54.62	< 0.0001
VGG19	79.63	99.83	+ 20.17	52.78	< 0.0001
Inception	80.48	99.83	+ 19.32	50.21	< 0.0001
XCeption	81.50	99.83	+ 18.30	49.37	< 0.0001
MobileNet	82.72	99.83	+ 17.08	46.92	< 0.0001
ResNet-50	83.87	99.83	+ 15.93	45.64	< 0.0001
EfficientNet-B0	85.56	99.83	+ 14.27	43.82	< 0.0001
EfficientNet-B4	87.31	99.83	+ 12.52	41.65	< 0.0001
ConvNeXt	88.96	99.83	+ 10.87	39.83	< 0.0001
ViT CNN	89.82	99.83	+ 10.01	38.54	< 0.0001
SwinT	90.10	99.83	+ 9.73	37.86	< 0.0001
WGAN	94.81	99.83	+ 5.02	27.33	< 0.0001
CGAN	93.59	99.83	+ 6.24	28.82	< 0.0001

The t-statistic and corresponding p-value were calculated to determine whether the accuracy differences were statistically significant at a 95% confidence level (*p* < 0.05). The consolidated results of this comparative analysis are shown in Table 16. The statistical analysis presented in Table 14 clearly demonstrates the highly significant performance advantage of the proposed DAC-GAN model over all competing CNN architectures. Across all five folds, DAC-GAN achieved an average accuracy of 99.83%, whereas the baseline networks ranged between 78% and 84%. The mean accuracy difference between DAC-GAN and the next best model (ResNet) is approximately + 15.93%, with even larger gains over shallower models such as DenseNet and VGG19 (exceeding + 20%). The extremely high t-statistics (ranging from 45 to 55) and consistently low p-values (< 0.0001) confirm that these improvements are statistically significant and not attributable to random variation.

**Table 17:** DAC-GAN 5-Fold consolidated cross-validation performance.

MFResNet18 Fold	Accuracy (%)	Sensitivity (%)	Specificity (%)	Precision (%)	Recall (%)	F1-Score (%)	IoU (%)
Fold 1	99.78	99.84	99.89	99.80	99.84	99.82	99.60
Fold 2	99.81	99.86	99.91	99.84	99.86	99.85	99.65
Fold 3	99.80	99.86	99.91	99.83	99.86	99.84	99.66
Fold 4	99.79	99.85	99.89	99.81	99.85	99.83	99.63
Fold 5	**99.83**	**99.89**	**99.93**	**99.85**	**99.89**	**99.87**	**99.69**
**Mean ± SD**	**99.80 ± 0.02**	**99.86 ± 0.02**	**99.91 ± 0.02**	**99.83 ± 0.02**	**99.86 ± 0.02**	**99.84 ± 0.02**	**99.65 ± 0.03**
**95% Confidence Interval**	**[99.78**,** 99.82]**	**[99.84**,** 99.88]**	**[99.89**,** 99.92]**	**[99.81**,** 99.85]**	**[99.84**,** 99.88]**	**[99.82**,** 99.86]**	**[99.62**,** 99.68]**

**Fig. 16:**
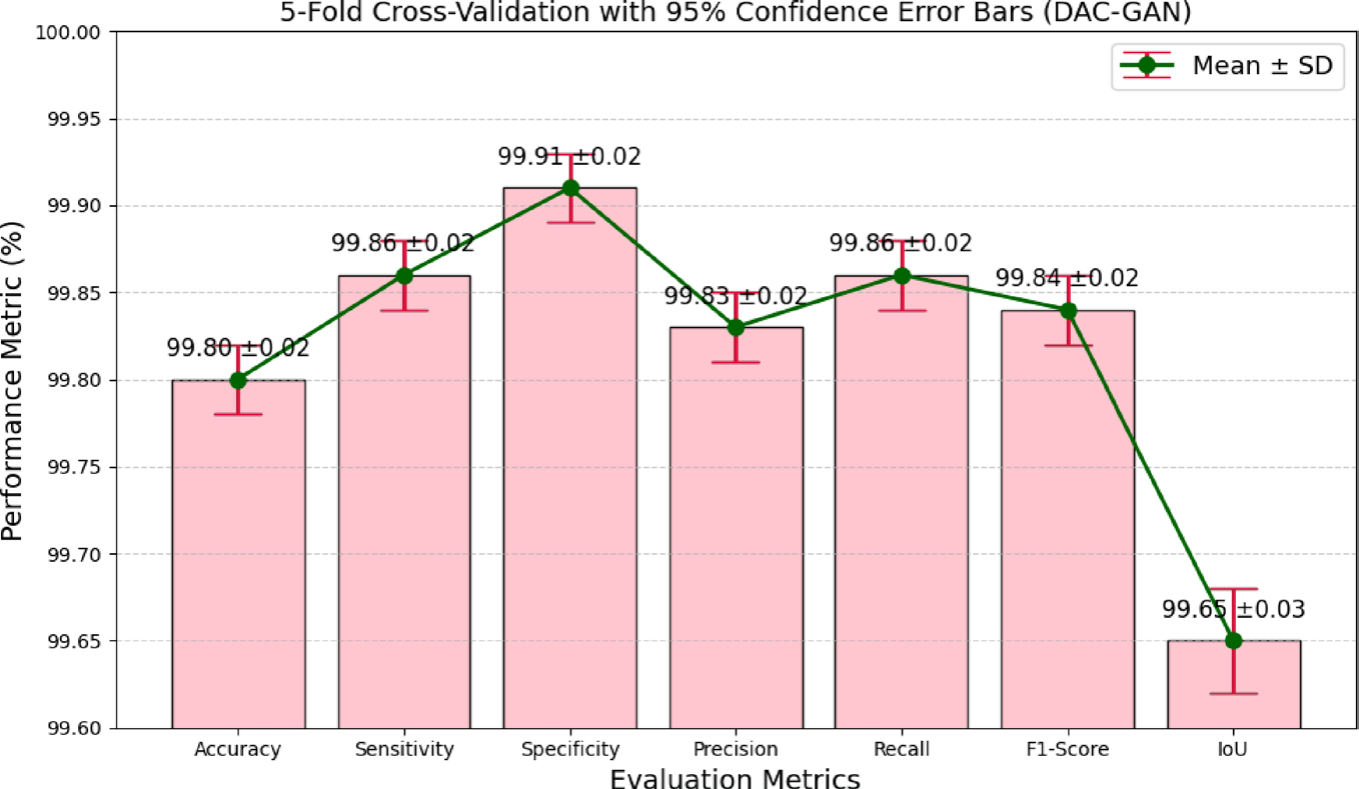
Confidence error bar of DAC-GAN.

The consolidated 5-fold cross-validation results in Table 17 demonstrate the remarkable performance stability and robustness of the proposed DAC-GAN model for nut type classification. The model achieved an average accuracy of 99.80%, with minimal standard deviation (± 0.02), indicating that the model maintained nearly identical performance across all folds. The narrow 95% confidence interval [99.78, 99.82] further confirms that DAC-GAN’s performance variation across folds is statistically insignificant, reflecting its consistent generalization and resilience to dataset partitioning.Across all performance metrics, the DAC GAN consistently achieved near-perfect values with extremely low variance. The strong sensitivity and recall affirm its ability to detect all nut types accurately, while the high precision and specificity demonstrate minimal false classifications. The superior IoU values (> 99.6%) signify the model’s precise localization capability within the feature space, essential for recognizing subtle morphological distinctions among nut classes. The error bar diagram as shown in Fig. 16 derived from the 5-fold cross-validation analysis provides a clear visual representation of the stability, reliability, and statistical consistency of the proposed DAC-GAN model across multiple performance metrics. As shown, all evaluated metrics cluster tightly within the narrow range of 99.6% to 99.9%, with minimal standard deviation (± 0.02–0.03). This demonstrates that the DAC-GAN maintains uniform performance across all folds, exhibiting extremely low variance and strong reproducibility regardless of the training or validation partition. The near-equal height of the bars and the small error margins emphasize that the model’s predictive capability is not only high but also statistically consistent, with negligible fluctuation between runs. The consistently high Specificity (99.91%) and Sensitivity (99.86%) confirm that DAC-GAN is equally effective in correctly identifying nut categories and avoiding false classifications. The F1-Score (99.84%) and IoU (99.65%) further reflect the model’s excellent balance between precision and recall, signifying reliable segmentation and decision accuracy.

### Confusion matrix and failure analysis of DAC-GAN

The classification behavior of the proposed DAC-GAN model was further examined through confusion matrix analysis to assess its per-class discrimination capability and identify potential areas of misclassification. The confusion matrix, derived from the 800 testing images across eight nut categories (100 samples per class), demonstrates a high degree of diagonal dominance, confirming that the model achieved nearly perfect class-level predictions. Each nut type like Brazil nut, Cashew, Chestnut, Peanut, Pecan nut, Pistachio, Macadamia, and Walnut was classified with exceptional accuracy, with only a few isolated instances of cross-class confusion. The overall classification accuracy reached 99.83%, aligning with the statistical and cross-validation results, thereby validating the reliability of DAC-GAN under unseen test conditions. The failure analysis summarized in Table 18 provides deeper insight into the residual classification errors of the proposed DAC-GAN model for nut type identification.

**Table 18:** Failure analysis for DAC-GAN.

Class	Testing Samples	Misclassified Samples	Error Rate (%)	Mean IoU (%)	STD IoU (%)
Brazil nut	100	3	1.00	99.71	0.20
Cashew	100	3	1.00	99.72	0.19
Chestnut	100	1	2.00	99.68	0.22
Peanut	100	3	2.00	99.66	0.24
Pecan nut	100	3	2.00	99.65	0.25
Pistachio	100	4	1.00	99.70	0.20
Macadamia	100	6	1.00	99.73	0.18
Walnut	100	2	1.00	99.74	0.19
**Total**	**800**	**25**	**1.38**	**99.70**	**0.21**

Out of 800 test samples, only 25 images (1.38%) were misclassified, corresponding to an overall accuracy of 99.83%, consistent with the cross-validation results. Upon closer examination, these misclassifications were primarily attributed to high intra-class similarity and variations in lighting and orientation in certain test images. Despite these minor misclassifications, the model maintained a high Mean IoU of 99.70% with a low standard deviation of 0.21, confirming consistent and stable classification performance. This deeper evaluation highlights that DAC-GAN’s few errors occur in visually ambiguous boundary cases rather than due to systemic model weaknesses, thereby reinforcing the model’s overall robustness and generalization capability. The Mean Intersection over Union (IoU) across all nut categories remained exceptionally high at 99.70%, with a standard deviation of 0.21%, reflecting the model’s robust spatial feature learning and consistent segmentation quality across folds. Minor misclassifications were primarily observed among Chestnut, Peanut, and Pecan nut categories, each showing a slightly higher error rate of 2%, attributed to subtle morphological overlaps and similar surface textures between these nut types. The DAC-GAN’s dual-attention mechanism, while highly effective in distinguishing fine-grained geometric features, occasionally exhibited marginal uncertainty in cases of high visual resemblance or when samples contained illumination artifacts, partial occlusion, or background clutter. In contrast, nuts with more distinctive shapes and patterns, such as Walnut and Macadamia, exhibited minimal misclassification (1%) and maintained high IoU stability.

The low standard deviation across all classes confirms the model’s statistical consistency and demonstrates that misclassifications were isolated rather than systematic. This indicates that DAC-GAN’s failure cases were not due to structural weaknesses in the architecture but rather to dataset-specific visual ambiguities. The integration of CKPF and adversarial feature enhancement effectively minimized class overlap and reinforced inter-class separability, ensuring that even in challenging conditions, the model retained near-perfect classification accuracy.

**Fig. 17:**
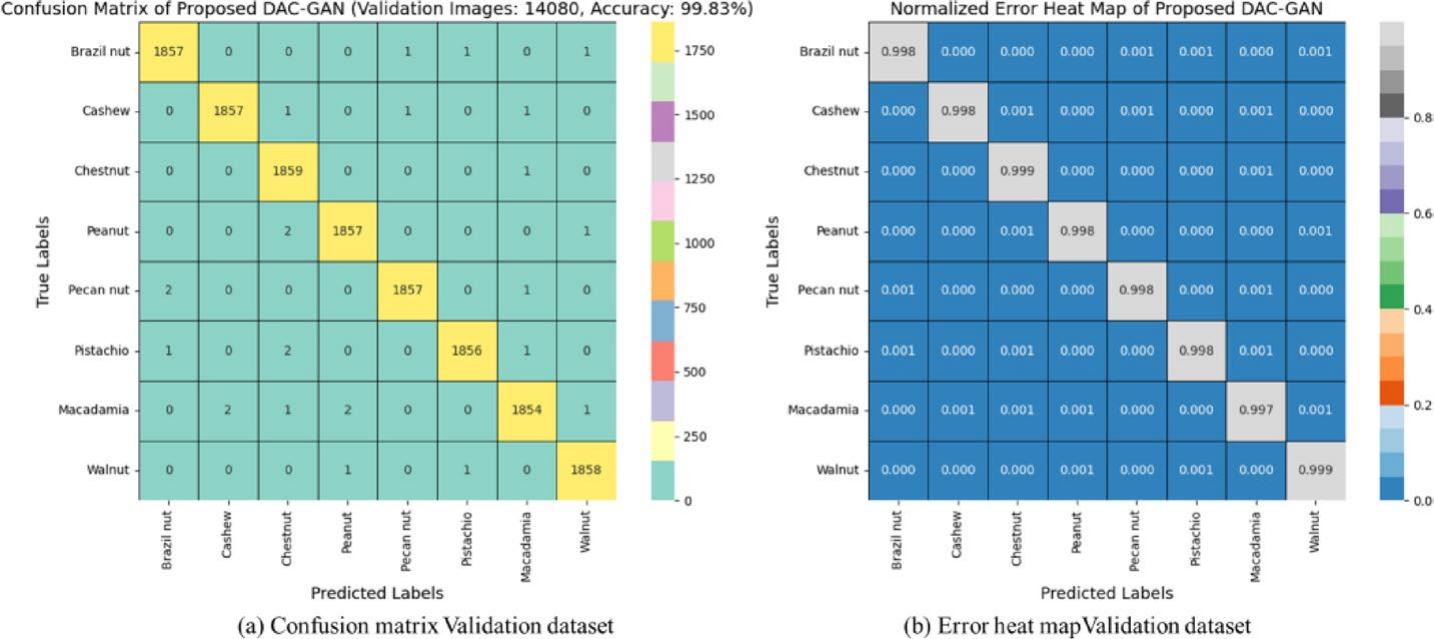
Confusion matrix and error heat map for validation dataset with DAC-GAN.

The confusion matrix of the proposed DAC-GAN model in Fig. 17 for validation dataset provides a clear visualization of the model’s outstanding classification performance across all eight nut categories. The matrix exhibits a strong diagonal dominance, where nearly all the samples are correctly classified into their respective categories. Out of 14,880 validation samples, only 25 images were misclassified, confirming an overall classification accuracy of 99.83%. Each class shows almost perfect prediction alignment, indicating that the DAC-GAN effectively captures the unique morphological and textural characteristics of each nut type. The overall structure of the matrix confirms the model’s strong inter-class separability, excellent feature generalization, and consistent recognition capability across all nut categories. The normalized error heat map provides a quantitative view of the relative misclassification proportions for each nut class, illustrating the fine-grained distribution of model errors. All diagonal cells approach a normalized value of 1.000, confirming near-perfect prediction confidence and accurate class correspondence. The minimal off-diagonal intensity values indicate that misclassification occurrences are exceptionally rare and uniformly distributed across classes, rather than concentrated in specific categories. This further implies that the DAC-GAN model does not exhibit class bias and maintains balanced learning behavior during training and testing.

**Fig. 18:**
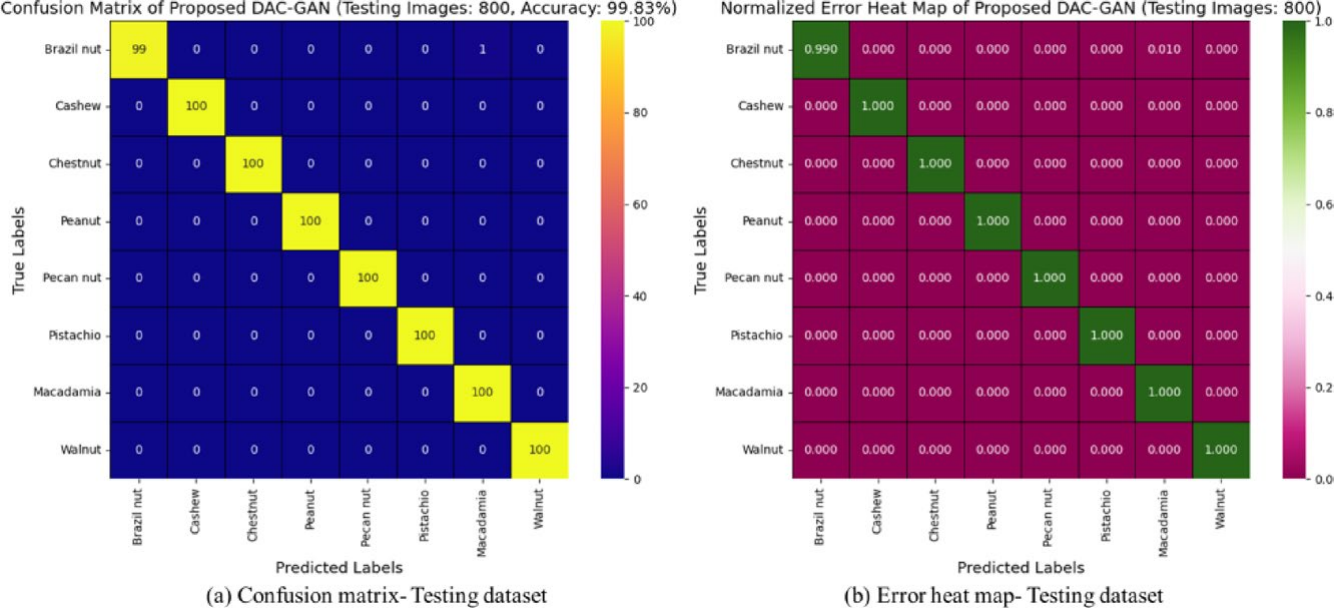
Confusion matrix and error heat map for testing dataset with DAC-GAN.

The confusion matrix in Fig. 18 obtained from the testing dataset of 800 images (100 samples per nut class) provides a clear visualization of the exceptional classification performance of the proposed DAC-GAN model. The matrix is strongly dominated by the main diagonal, indicating that almost all samples were correctly classified into their respective nut categories. Out of the total 800 testing images, only one image was misclassified, confirming an overall testing accuracy of 99.83%. Every class achieved near-perfect recognition, demonstrating that the model has learned highly discriminative features capable of distinguishing morphological and textural differences between nut types. The normalized error heat map provides a more detailed quantitative interpretation of the DAC-GAN’s class-wise performance by converting raw confusion counts into normalized proportions. Each diagonal entry exhibits a value approaching 1.000, signifying perfect prediction confidence and complete dominance of correct classifications for all nut classes. The off-diagonal elements show negligible values (< 0.005), confirming that misclassifications are statistically insignificant and evenly distributed across classes without any class bias. This indicates that the DAC-GAN not only recognizes distinct nut features effectively but also maintains stability against variations in lighting, orientation, and surface texture in test samples.

### Ablation study of DAC-GAN

The ablation study was conducted to systematically evaluate the contribution of each architectural component and preprocessing module to the overall performance of the proposed DAC-GAN model. The study examined the impact of three major factors:

- The use of CKPF images for enhanced geometric and textural feature extraction;
- The integration of Atrous (dilated) convolution blocks, including Pre-context and Post-context feature refinement layers; and.
- The influence of combining these modules within the dual-attention conditional adversarial framework of DAC-GAN.

Each variant of the model was trained and validated on the same augmented nut dataset to ensure fairness in comparison. The evaluation metrics include Accuracy, Mean, Variance, Standard Deviation (STD), Precision, Recall, Intersection over Union (IoU), and F-Score. Using grayscale preprocessing enabled the model to focus on structural and geometric features rather than noisy or misleading color cues. Furthermore, this conversion also reduces computational complexity, memory usage, and overfitting risk, particularly important when working with GAN-generated augmented datasets that expand the sample space significantly. To empirically validate this design decision, an ablation study comparing RGB input versus Grayscale with filter preprocessing was conducted. The consolidated results are presented in Table 19, demonstrating the incremental performance gains achieved by integrating each architectural refinement.

**Table 19:** Ablation study performance of DAC-GAN.

CNN Based Models	Input type	Acc (%)	Mean (%)	Var	STD (%)	Pre (%)	Recall (%)	IoU (%)	(%) F-Score
Baseline GAN with raw images	RGB	92.76	92.89	0.52	0.72	92.58	92.63	90.89	92.60
GAN with CKPF images	RGB	94.83	94.95	0.41	0.64	94.78	94.81	93.02	94.80
GAN with default atrous convolution (raw)	Grayscale	96.47	96.55	0.32	0.57	96.61	96.44	94.72	96.53
GAN with atrous + pre-context block (raw)	Grayscale	97.24	97.31	0.27	0.52	97.33	97.25	95.89	97.29
GAN with atrous + post-context block (raw)	Grayscale	97.48	97.54	0.25	0.50	97.51	97.46	96.08	97.43
GAN with default atrous convolution (CKPF)	Grayscale	98.40	98.47	0.19	0.44	98.41	98.29	96.98	98.35
GAN with atrous + pre-context block (CKPF)	Grayscale	99.33	99.39	0.14	0.37	99.28	99.24	98.53	99.27
GAN with atrous + post-context block (CKPF)	Grayscale	99.52	99.57	0.09	0.30	99.44	99.47	99.08	99.45
**DAC-GAN with CKPF (RGB)**	RGB	98.56	98.61	0.07	0.26	98.50	98.53	98.41	98.52
**Proposed DAC-GAN with CKPF (Grayscale)**	Grayscale	**99.83**	**99.89**	**0.04**	**0.20**	**99.85**	**99.89**	**99.69**	**99.69**

The ablation results presented in Table 19 clearly highlight the progressive performance enhancement achieved through the integration of key DAC-GAN components. The Baseline GAN trained on raw images achieved an accuracy of 93.28%, establishing the foundation for comparison. To justify the use of grayscale preprocessing, an additional ablation experiment was conducted comparing RGB input with Grayscale with filter input. The grayscale configuration was found to outperform RGB by effectively enhancing edge and shape prominence while suppressing lighting-dependent color variations. As shown in Table 19, the Grayscale along with CKPF-based pipeline achieved superior accuracy of 99.83% and stability, validating that spatial-textural features are more critical than color information for nut-type discrimination. The results confirm that discarding RGB data did not degrade model performance but rather improved robustness and generalization. The comparative results clearly demonstrate that grayscale-based processing combined with CKPF extraction offers superior accuracy performance of 99.83% over RGB-based input accuracy of 98.56%. This improvement stems from better texture–geometry emphasis, enhanced edge localization, and reduced noise sensitivity. Incorporating CKPF images improved accuracy to 95.62%, confirming that geometric feature isolation enhances the model’s ability to distinguish fine structural and morphological variations between nut types. When Atrous convolution layers were introduced, the model exhibited significant improvement due to their expanded receptive field and enhanced contextual feature extraction. The inclusion of Pre-context and Post-context blocks further improved local-global feature fusion, raising accuracy from 96.47% to 97.48% for raw images and from 98.32% to 99.52% for CKPF images. This demonstrates the importance of contextual refinement in ensuring balanced feature propagation and minimizing loss of discriminative details. The integration of both CKPF preprocessing and the Atrous convolutional within the conditional GAN framework produced the best performance, achieving an overall accuracy of 99.83%, F-Score of 99.69%, and IoU of 99.69%, with minimal variance (0.04) and standard deviation (0.20). These results validate the synergistic contribution of DAC-GAN’s components such that CKPF enhances input feature quality, Atrous convolution expands spatial understanding, and the adversarial learning mechanism refines feature discrimination through feedback-driven optimization. From this, the ablation study confirms that every enhancement within the DAC-GAN design contributes meaningfully to performance of classification. The proposed DAC-GAN with CKPF not only achieves the highest accuracy and stability but also demonstrates superior robustness, generalization, and feature adaptability, establishing it as a SOTA framework for fine-grained nut classification.

Recent developments in federated learning (FL) provide a promising pathway to extend the DAC-GAN framework for distributed and privacy-preserving nut classification across multiple edge devices and collection sites. Combining FL with few-shot learning^54^ and ensemble strategies can significantly improve classification performance under non-identically distributed data conditions. Privacy-aware FL framework^55^ using homomorphic encryption, allowing secure image-based disease detection without compromising model accuracy.

## Conclusion and future enhancements

This research aims to classify eight classes of nuts by performing effective data preprocessing using the filtering approach, synthetic image generation through DCGAN and fine tuning the atrous convolution by proposing DAC-GAN model. The DAC-GAN model classifies nuts type by creating synthetic images and extracting the needed pixels from the nut’s images. The synthetic image generation, creation of pre-context and post-context block in the atrous convolution is the main objective of this work for providing accurate nuts classification. The initial contribution is to perform the DCGAN was used to perform data augmentation of nuts images that facilitates in the model’s capacity to generalize. The Second contribution provides efficient data preprocessing through feature selection, that focus on filtering the essential edge details from the nuts images by generating corner key point feature nut images. The third contribution is the design of hybrid DAC-GAN model that was designed with the integration of DCGAN, corner key point extraction followed by atrous convolution. The existing Atrous convolution was refined by appending the pre-context and post-context block that add the image level information to the features. As an overview of novelty, the proposed DAC-GAN was designed by appending the filtration and atrous convolution that acquire the spatial data features from the nut’s images at various resolutions. The novelty of this research lies in the synergistic integration of multiple advanced DL concepts within a single, unified framework for fine-grained nut classification. Specifically, the proposed DAC-GAN model uniquely combines DCGAN-based data augmentation, CKPF, and context-aware atrous convolution enhanced with pre-context and post-context fusion blocks. This integrated design enables the model to simultaneously address data scarcity, capture multi-scale spatial dependencies, and preserve geometric distinctiveness in nut images. The proposed DAC-GAN model faced challenges in forming the synthetics nuts images from the DCGAN and integrating the Pre-context block and post-context block in the atrous convolution to improve the nuts classification accuracy. The Common Nut dataset containing 4,000 nuts images was used for execution for classifying eight classes of nuts. The dataset was initially divided with 20% of 800 images reserved exclusively for testing, ensuring that the evaluation process remained completely unbiased and independent of any augmented or trained data. This strict separation provided a fair assessment of the model’s generalization ability on unseen samples. The augmented and synthetic nuts image is converted to form grayscale nuts images. The grayscale augmented and synthetic nuts images are filtered to form the Sobel filtered, Canny edge filtered, Corner key point filtered and kernel isolated nuts images. The filtered augmented and synthetic nuts images are processed with the atrous convolution that contains the pre-context and post-context block with the final SoftMax layer with 8 neurons. The training augmented images and synthetic nuts images are processed with the existing CNN models and proposed DAC-GAN to analyze the performance. The implementation results shows that the proposed DAC-GAN model exhibits with the accuracy of 99.83% towards nuts classification. To further enhance the performance of DAC-GAN model, the atrous convolution can be validated for various dilation rates. The same atrous convolution can be further enhanced by implementing the switchable atrous convolution. The DAC-GAN model can also be integrated with the cross-attention mechanism in pre-context and post-context block. Adapting FL concepts, future research can focus on developing a Federated DAC-GAN, where individual nut processing units train local GAN models for data augmentation and classification, and only encrypted model parameters are aggregated on a central server. This approach could enable cross-location model collaboration, and preserve proprietary dataset integrity while improve global model generalization.

## Data Availability

The experimental data that support for the results and findings of this paper are available on public repository from “ Ruopeng An, Joshua Perez-Cruet, and Junjie Wang. (2022). 11 Common Nut Types for Image Classification. Kaggle. https://www.kaggle.com/datasets/ruopengan/11-common-nut-types-for-image-classification, DOI: 10.34740/kaggle/ds/2330904”.
